# Efficacy of adjunctive antibiotics compared to non-antibiotic therapies following mechanical debridement for peri-implantitis: A systematic review and meta-analysis of randomized controlled trials

**DOI:** 10.1371/journal.pone.0352311

**Published:** 2026-06-25

**Authors:** Caiyun Liu, Hongtao Wang, Yulan Li, Zhilong Huang, Jian Sun, Han Cai

**Affiliations:** 1 Yichang Central People’s Hospital, The First College of Clinical Medical Science, Three Gorges University, Yichang, China; 2 Guangzhou Medical University, Guangzhou, China; 3 Renmin Hospital of Wuhan University, Wuhan, China; UCLA School of Dentistry, UNITED STATES OF AMERICA

## Abstract

**Objective:**

To evaluate the efficacy of antibiotic therapy as an adjunct to mechanical debridement in peri-implantitis.

**Method:**

PubMed, Embase, Web of Science, the Cochrane Library, and Scopus were searched (inception to 15 January, 2026). Additionally, clinical trials were searched on ClinicalTrials.gov. Meta-analyses were performed to evaluate bone level (BL), bleeding on probing (BOP), probing pocket depth (PPD), clinical attachment level (CAL), modified plaque index (mPLI), plaque score (PS).

**Results:**

Twenty-two RCTs were included. Adjunctive antibiotics with mechanical debridement demonstrated significant advantages in improving PPD (WMD = −0.69; 95% CI: [−1.04, −0.33]; P = 0.0001; heterogeneity:I^2^ = 91%, P < 0.00001), CAL (WMD = −0.55; 95% CI: [−1.07, −0.04]; P = 0.04; heterogeneity:I^2^ = 95%, P < 0.00001), PS (WMD = −4.07; 95% CI: [−6.80, −1.33]; P = 0.004; heterogeneity:I^2^ = 0%, P = 0.94), and BOP (WMD = −12.45; 95% CI: [−24.13, −0.77]; P = 0.04; heterogeneity:I^2^ = 94%, P < 0.00001). In the subgroup analysis comparing mechanical debridement plus antibiotics versus mechanical debridement alone, statistically significant improvements were observed for CAL (WMD = −0.84; 95% CI: [−1.55, −0.13]; P = 0.02) and PS (WMD = −3.94; 95% CI: [−7.14, −0.74]; P = 0.02), confirming clinical necessity of antibiotics as an adjunct to debridement. In the comparison of local versus systemic antibiotic administration, comparable efficacy in reducing PPD was observed for both local (WMD = −0.68; 95% CI: [−1.14, −0.22]; P = 0.004) and systemic (WMD = −0.70; 95% CI: [−1.25, −0.15]; P = 0.01) routes, with no significant difference between subgroups (P = 0.95, I² = 0%). For PS, local antibiotics (WMD = −6.57; 95% CI: [−11.10, −2.04]; P = 0.004) demonstrated significantly greater improvement compared to systemic antibiotics (WMD = −2.63; 95% CI: [−6.07, 0.80]; P = 0.13).

**Conclusion:**

As an adjunct to mechanical debridement, antibiotics may provide modest benefits for peri-implantitis, demonstrating efficacy in improving probing pocket depth, clinical attachment level, plaque score, bleeding on probing, and other parameters related to soft tissue health and plaque control.

**PROSPERO:**

CRD420261328879. Available from https://www.crd.york.ac.uk/PROSPERO/view/CRD420261328879.

## Introduction

Peri-implant diseases are common biological complications in clinical practice, defined as inflammatory lesions affecting the soft and hard tissues surrounding dental implants. They are primarily categorized into peri-implant mucositis (PM) and peri-implantitis (PI) [1]. PM refers to inflammation confined to the peri-implant mucosa without supporting bone loss. In contrast, PI involves both mucosal inflammation and progressive bone loss, clinically presenting with deep probing pocket depth (PPD), bleeding on probing (BoP), and suppuration on probing (SoP) [2–5]. Although the overall survival rate of dental implants remains high [6], the prevalence of peri-implant diseases is substantial. According to the case-definition criteria proposed by Sanz and Chapple [7], a recent systematic review reported a prevalence of 18.5% at the patient level and 12.8% at the implant level, with the risk increasing over time [8]. The primary etiology is infection driven by plaque biofilm accumulation, where the surface roughness and threaded architecture of the implant provide an ideal microenvironment for pathogenic bacterial colonization. Consequently, the core therapeutic objective is the thorough decontamination of the implant surface and effective infection control [9–12].

However, the clinical management of peri-implantitis faces significant challenges. Due to anatomical limitations imposed by implant threads, surface topography, and prosthesis design, instruments often fail to adequately access the lesion areas. As a result, simple mechanical debridement is insufficient for the complete eradication of deep-seated biofilms, leading to limited therapeutic efficacy and unresolved inflammation [13]. Therefore, adjuvant antimicrobial therapies are widely utilized in clinical practice. These mainly include the local administration of sustained-release antibiotics (e.g., minocycline microspheres, doxycycline gel), systemic antibiotics (e.g., azithromycin, or metronidazole combined with amoxicillin), chlorhexidine gel, and photodynamic therapy (PDT) [14]. Among these, local antibiotic delivery possesses a theoretical advantage by maintaining a high drug concentration directly at the infection site.

Despite their widespread use, the clinical value of antibiotics as an adjunct to mechanical debridement remains highly controversial, as evidenced by conflicting conclusions from recent meta-analyses. Over the past years (2022–2025), several high-level systematic reviews have attempted to address this issue but have yielded inconsistent findings. For instance, a meta-analysis(Baus-Domínguez M et al. 2023) focusing specifically on surgical treatment concluded that local antibiotics significantly improved PPD, whereas systemic antibiotics provided no significant adjunctive benefit [15]. Conversely, a meta-analysis published in 2024 that pooled both non-surgical and surgical interventions reported that systemic antibiotics significantly enhanced PPD reduction and radiographic bone gain [16]. Furthermore, meta-analyses strictly addressing non-surgical treatments (updated in 2022 and 2025) suggested only marginal overall benefits from adjunctive antibiotics, coupled with substantial heterogeneity [17,18].

These discrepancies largely stem from critical methodological limitations in the existing literature. First, previous meta-analyses frequently mixed different therapeutic contexts (e.g., pooling non-surgical and surgical approaches) and disease boundaries (occasionally including peri-implant mucositis), obscuring the specific efficacy of antibiotics for true peri-implantitis. Second, and most importantly, previous studies rarely defined a strictly “active” control group. They typically compared “mechanical debridement plus antibiotics” against “mechanical debridement alone” or placebo, failing to account for the fact that clinicians routinely use other non-antibiotic adjuncts (such as PDT or antiseptics). Finally, while local and systemic antibiotics differ fundamentally in pharmacokinetics and antimicrobial spectra, prior reviews often failed to isolate and directly compare these two distinct routes as primary subgroups.

Given the high degree of inconsistency in the existing evidence and the aforementioned methodological gaps, there is a pressing need for a refined quantitative synthesis. Therefore, the aim of this systematic review and meta-analysis was to strictly evaluate the efficacy of antibiotics as an adjunct to mechanical debridement for peri-implantitis compared against other non-antibiotic adjunctive treatments. Furthermore, we conducted predefined subgroup analyses to delineate the distinct efficacy of local versus systemic antibiotic administration, thereby providing more precise and reliable evidence-based guidance for clinical decision-making.

## Methods

### Study design

This meta-analysis was conducted in accordance with the 2020 Preferred Reporting Items for Systematic Reviews and Meta-Analyses (PRISMA) guidelines [19] and registered in the PROSPERO database (CRD420261328879).

### Search strategy

A systematic literature search was performed in PubMed, Embase, Web of Science, Scopus, and the Cochrane Library from database inception to January 15, 2026. Relevant clinical trials were also identified through ClinicalTrials.gov. The search strategy was developed in accordance with the PICOS (Population, Intervention, Comparison, Outcomes, Study design) framework and incorporated a combination of MeSH terms and free-text keywords. No language restrictions were applied.

The specific search terms included “Peri-Implantitis”, and “Antibiotic”. Detailed search records are provided in the Supplementary Materials.

### Inclusion and exclusion criteria

Inclusion criteria were as follows: (1) Patients diagnosed with peri-implantitis(standardized diagnostic criteria: Bone loss ≥2 mm + PPD ≥ 5 mm + BOP positive); (2) Patients in the intervention group received antibiotics as an adjunct to mechanical debridement (Antibiotic group); (3) Patients in the controlled group received other treatments (Non-antibiotic group); (4) At least the following results should be reported: bone level (BL), bleeding on probing (BOP), probing pocket depth (PPD),clinical attachment level (CAL), modified plaque index (mPLI), plaque score (PS); (4) Study types: randomized controlled trials.

The exclusion criteria are as follows: (1) Other types of articles, such as case reports, conference, publications, thesis, letters, comments, reviews, meta-analyses, editorials, protocols, etc; (2) Other diseases; (3) Not relevant; (4) The outcomes were not reported.

Given the relatively small number of studies utilizing active comparator groups, we pooled studies with heterogeneous control interventions–namely mechanical debridement alone, debridement plus probiotics, and debridement plus photodynamic therapy (PDT)–within the primary comparison of ‘antibiotics plus debridement versus controls’. This decision was based on several considerations. First, all control interventions represent non-antibiotic adjunctive approaches that aim to enhance the effects of mechanical debridement; their common comparator status allowed us to maximize the power for detecting an overall treatment effect of adjunctive antibiotics. Second, both probiotics and PDT are mechanistically distinct from systemic/local antibiotics and are not expected to exert the same magnitude or direction of antimicrobial effect. Third, to empirically address the concern of increased heterogeneity, we conducted a pre-specified sensitivity analysis excluding trials with active controls (probiotics or PDT), thereby evaluating the impact of control group variability on the robustness of the pooled estimates.

### Selection of articles

Literature screening was managed using EndNote (Version 20; Clarivate Analytics) to identify and remove duplicate records. Two independent reviewers initially screened the titles and abstracts of the remaining records for relevance. For studies undergoing full-text review, supplementary materials were consulted if relevant efficacy data were not reported in the main text. Additionally, ClinicalTrials.gov was searched using trial registration identifiers (e.g., NCT numbers) to retrieve any published outcomes. Disagreements between the two reviewers were resolved through consensus, with consultation of a third author as an arbiter when necessary.

### Data extraction

The data were extracted independently by two reviewers using standardized forms. The extracted information included: (1) Basic study characteristics, including region, and publication years; (2) Baseline demographics and clinical characteristics of participants, including sample size, age, treatment regimens, and follow-up duration; (3) Efficacy outcomes. Any discrepancies were resolved through consultation with a third investigator.

### Quality assessment

Two independent reviewers assessed the quality of the included trials. Specifically, the quality of randomized controlled trials (RCTs) was evaluated using the Cochrane Risk of Bias Tool for Randomized Trials [20,21]. Any discrepancies in the assessment process were resolved by group consensus.

### Statistical analysis

Statistical analyses were performed using Cochrane Review Manager 5.3, R software (Version 4.5.2), RStudio (2026.01) and Gradeprofiler. The comparison of continuous variables was conducted using the weighted mean difference (WMD) along with a 95% confidence interval (CI). The relative ratio (RR) with a 95% CI was employed to compare binary variables. The medians and interquartile ranges of continuous data were transformed into the mean and standard deviation. When studies reported data as medians and interquartile ranges (IQR), we estimated the mean and standard deviation (SD) using the method of Wan et al. [22]. For studies with sample size n ≤ 25, mean ≈ (Q1 + median + Q3)/3 and SD ≈ IQR/1.35. For n > 25, mean was approximated similarly, and SD was calculated as IQR/1.35 for n ≤ 70, and for n > 70 SD ≈ IQR/1.35. In all cases, we assumed the underlying data followed a normal distribution. The statistical heterogeneity among the included studies was assessed using the Cochrane’s Q test and the I^2^ index. Random-effects models employing the DerSimonian-Laird technique were utilized in considerable heterogeneity (Q tests, P < 0.05; I^2^ > 50%); otherwise, a fixed model will be adopted. It is worth noting that the random model is not universally applied; instead, its use is determined based on whether the heterogeneity exceeds 50%. For outcomes with significant heterogeneity (I^2^ > 50%), univariate meta-regression was applied to identify potential heterogeneous sources, with covariates covering study region, participant age, sample size, male proportion, follow-up time and publication year. Meta-regression was limited to exploring heterogeneity, not causal inference. Meta-regression would be replaced by subgroup and sensitivity analyses when the number of included studies was less than 10 (k < 10). P values <0.05 were considered statistically significant.

## Results

### Search results

**Fig 1:**
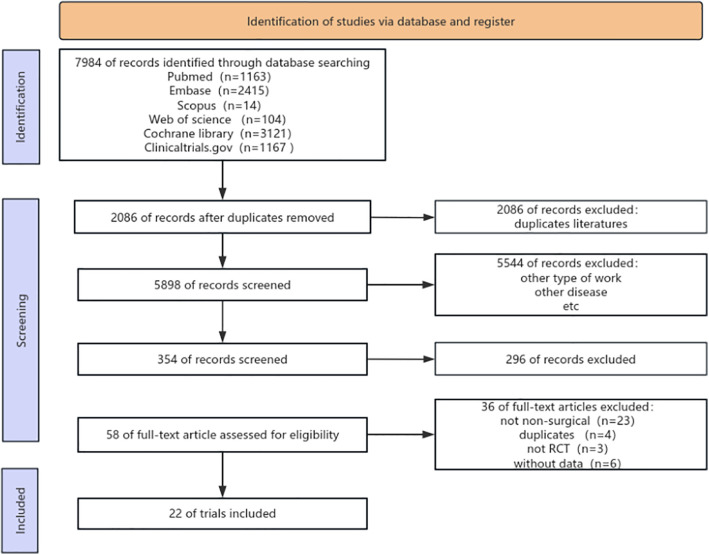
PRISMA flow diagram.

The literature selection process is illustrated in Fig 1. A total of 7,984 records were initially identified. Following the removal of 2,086 duplicates—primarily using the automatic deduplication function in EndNote—5,898 records remained. After excluding ineligible publication types (e.g., reviews, protocols, case reports, animal studies, meta-analyses, studies on other diseases, and retrospective studies), 5,544 irrelevant records were discarded. Subsequent manual screening of titles and abstracts led to the exclusion of an additional 296 articles. Ultimately, 22 studies met the eligibility criteria after full-text review, consultation of supplementary materials, and verification of trial registration numbers.

### Patient characteristics

This meta-analysis included 22 RCTs, with a total of 1028 patients enrolled. Specifically, 487 patients were allocated to the antibiotic group and 541 to the non-antibiotic group. Across all included trials, patients were diagnosed with peri-implantitis, and received the treatment of mechanical debridement. The antibiotics used in the intervention group were minocycline, amoxicillin, metronidazole, azithromycin and doxycycline. Detailed baseline characteristics of the included trials and participants are presented in Table 1.

**Table 1:** Baseline characteristics of studies and participants included.

Author，year	Country	Patients	Age (mean,sd)	Intervention group	Control group	Follow- up time (month)
Na Wei 2020	China	86	27.13 ± 5.86	mechanical debridement+local minocycline hydrochloride	mechanical debridement+iodine glycerin	1
De Waal 2021	Netherlands	62	I:60.0 ± 10.4 C:53.5 ± 11.2	mechanical debridement+systemic amoxicillin and metronidazole	mechanical debridement+ chlorhexidine	3
Kumararama 2024	India	60	18–65 years	mechanical debridement+systemic amoxicillin	mechanical debridement mechanical debridement+probiotic	3
Faramarzi 2015	Iran	69	I:48.4 ± 2.9 C:MD 45.6 ± 2.9 C:EMD 49.9 ± 2.9	mechanical debridement+local micro-spherical minocycline	mechanical debridement mechanical debridement+enamel matrix derivative	3
Al Deeb 2020	Saudi Arabia	45	I:53.8 ± 0.7 C:MD 49.2 ± 0.13 C:aPDT 52.6 ± 0.9	mechanical debridement+local azithromycin	mechanical debridement mechanical debridement +antimicrobial photodynamic therapy	3
Park 2021	Korea	118	61.1 ± 9.25	mechanical debridement+local metronidazole-minocycline ointment mechanical debridement+local minocycline ointment	mechanical debridement	3
Polymeri 2022	Netherlands	37	I:58.3 ± 13.9 C:60.8 ± 14.8	mechanical debridement+systemic amoxicillin and metronidazole	mechanical debridement	3
Alhumaidan 2022	Saudi Arabia	48	Smoker: I:56.1 ± 4.7 C:52.2 ± 2.4 No-smoker: I:52.8 ± 3.1 C:55.1 ± 1.7	mechanical debridement+local minocycline	mechanical debridement	6
Ahmed 2020	Malaysia	60	I:51.4 ± 6.7 C:MD 50.7 ± 5.9 C:aPDT 48.9 ± 4.5	mechanical debridement+local metronidazole and amoxicillin	mechanical debridement mechanical debridement +antimicrobial photodynamic therapy	6
Alqahtani 2022	Saudi Arabia	42	I:45.3 ± 4.9 C:MD 46.1 ± 3.7 C:P 45.2 ± 5.1	mechanical debridement+systemic amoxicillin	mechanical debridement mechanical debridement+probiotic	6
Chitsazi 2022	Iran	32	I:31.81 ± 4.94 C:32.75 ± 5.07	mechanical debridement+local sterile topical tetracycline ophthalmic ointment	mechanical debridement	6
Gershenfeld 2018	Australia	17	I:59.7 ± 13.1 C:64.4 ± 8.5	mechanical debridement+systemic azithromycin	mechanical debridement	6
Hallstro¨m 2012	Sweden	48	I:54.6 ± 18.2 C:54.6 ± 19.8	mechanical debridement+systemic azithromax	mechanical debridement	6
Scha¨ r 2012	Switzerland	40	I:57 ± 11.5 C:59 ± 12.75	mechanical debridement+local minocycline	mechanical debridement+photodynamic therapy	6
Gomi 2015	Japan	20	67.6 ± 5.3	mechanical debridement+systemic azithromycin	mechanical debridement	12
Almohareb 2020	Saudi Arabia	40	I:50.9 ± 6.3 C: 51.7 ± 7.5	mechanical debridement+systemic amoxicillin and metronidazole	mechanical debridement +antimicrobial photodynamic therapy	12
Bassetti 2013	Switzerland	40	I:57 ± 11.5 C:59 ± 12.75	mechanical debridement+local minocycline	mechanical debridement +antimicrobial photodynamic therapy	12
Blanco 2021	Spain	32	I:58.31 ± 3.43 C:60.74 ± 2.69	mechanical debridement+systemic metronidazole	mechanical debridement	12
Buchter 2004	Germany	28	I:54 ± 9 C:56 ± 10	mechanical debridement+local doxycycline	mechanical debridement	12
Renvert 2006	Sweden	32	I:65.6 ± 8.6 C:61.1 ± 8.6	mechanical debridement+local minocycline	mechanical debridement+chlorhexidine	12
Renvert 2008	Sweden	32	41 - 75 years	mechanical debridement+local minocycline	mechanical debridement+chlorhexidine	12
Shibli 2019	Brazil	40	58.5 ± 11.1	mechanical debridement+systemic metronidazole and amoxicillin	mechanical debridement	12

I:Intervention group, using antibiotics.

C:Control group, not using antibiotics(MD:mechanical debridement, EMD:enamel matrix derivative, aPDT:antimicrobial photodynamic therapy, P:probiotic).

### Risk of Bias and Evidence Certainty Assessment

The risk of bias in the included randomized trials was assessed using the Cochrane Risk-of-Bias tool. A summary of the assessment is presented in S1 Fig in S1 File.

The certainty of evidence for specific outcomes was evaluated using the GRADEpro GDT framework. The overall certainty of evidence regarding probing pocket depth (PPD) was assessed as high (S2 Fig in S1 File). Detailed GRADE profiles are provided in the Supplementary Materials.

### Probing pocket depth (PPD)

**Fig 2:**
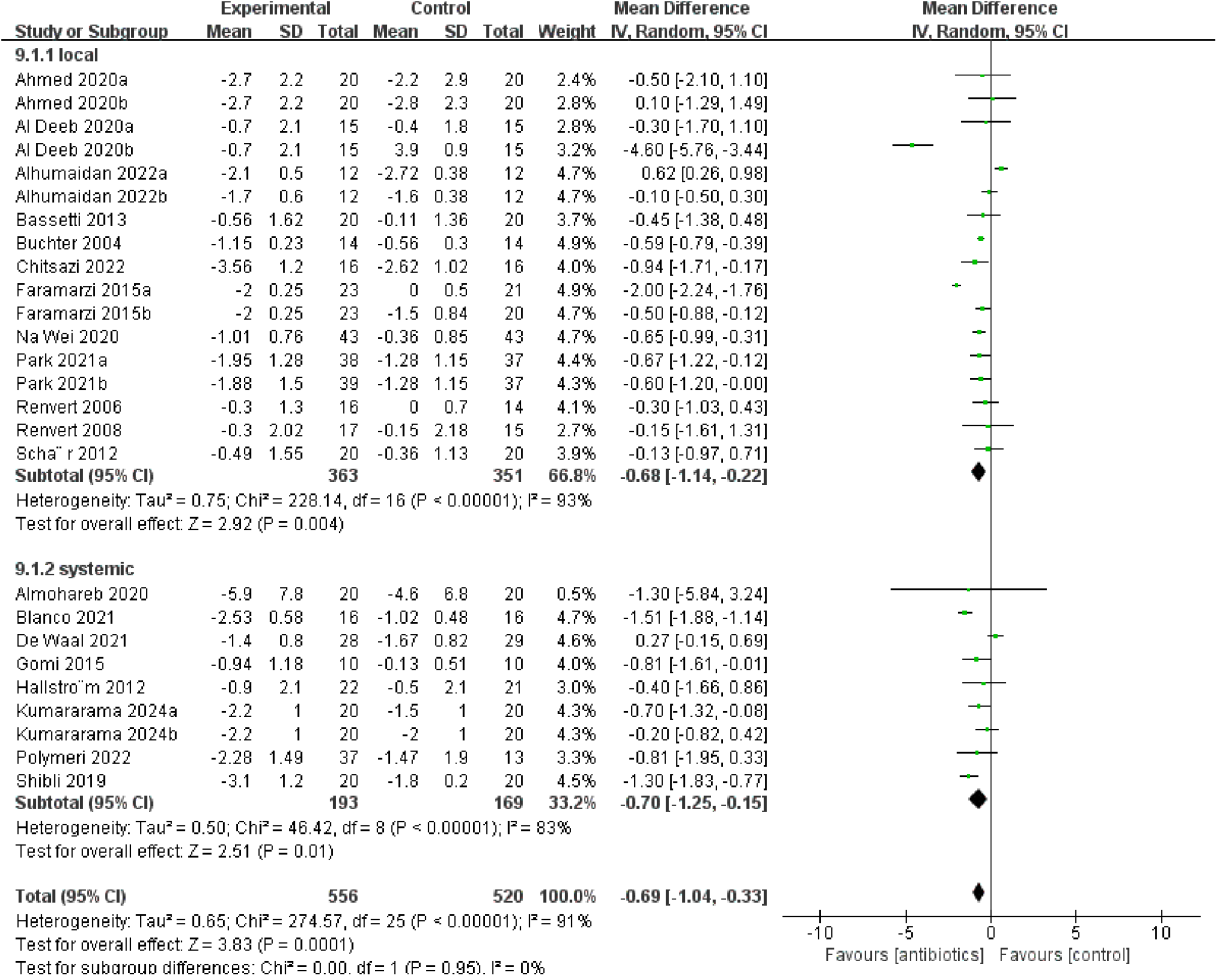
Forest plot of probing pocket depth (PPD). a and b are different groups of the same trial. local: local antibiotics；systemic：systemic antibiotics. Experimental group: antibiotics plus mechanical debridement. Control group: mechanical debridement; mechanical debridement plus enamel matrix derivative; mechanical debridement plus antimicrobial photodynamic therapy; mechanical debridement plus probiotic. NOTE:PPD: mean difference in mm.

Twenty trials assessed changes in PPD. Overall, adjunctive antibiotics significantly reduced PPD compared to controls (WMD = −0.69, 95% CI: [−1.04, −0.33], P = 0.0001; heterogeneity:I^2^ = 91%, P < 0.00001;Fig 2;Hartung–Knapp–Sidik–Jonkman:MD = −0.686, P = 0.0009). Subgroup analysis demonstrated significant reductions for both local (WMD = −0.68, 95% CI: [−1.14, −0.22], P = 0.004; heterogeneity:I^2^ = 93%, P < 0.00001;) and systemic (WMD = −0.70, 95% CI: [−1.25, −0.15], P = 0.01; heterogeneity:I^2^ = 83%, P < 0.00001;) antibiotic administration(Supplementary Materials S6 Fig in S1 File). Furthermore, in the comparison of mechanical debridement plus antibiotics versus mechanical debridement alone, the adjunctive use of antibiotics yielded superior therapeutic outcomes. Subgroup analyses stratified by antibiotic type showed significant differences in subgroups containing only one study, while no statistically significant differences were detected in subgroups with two or more studies(Supplementary Materials S12 Fig in S1 File). Subgroup analyses based on follow-up duration revealed statistically significant differences at the 3-month and 12-month follow-up time points(Supplementary Materials S18 Fig in S1 File).

### Bleeding on probing (BOP)

**Fig 3:**
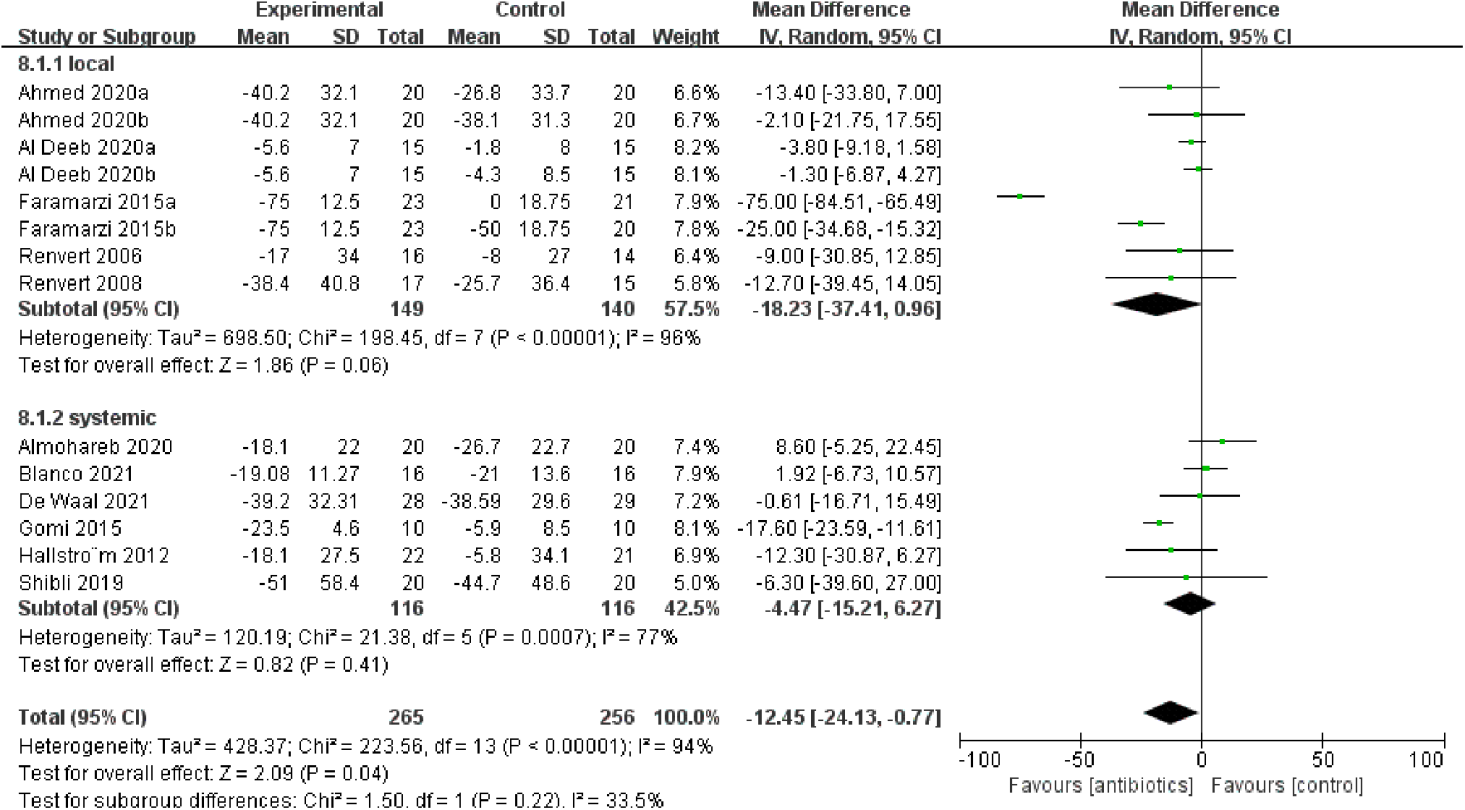
Forest plot of bleeding on probing (BOP). a and b are different groups of the same trial. local: local antibiotics；systemic：systemic antibiotics. Experimental group: antibiotics plus mechanical debridement. Control group: mechanical debridement; mechanical debridement plus enamel matrix derivative; mechanical debridement plus antimicrobial photodynamic therapy; mechanical debridement plus probiotic. NOTE:BOP: mean difference in percentage of positive sites (%).

Eleven trials reported changes in BOP. As shown in Fig 3, the experimental group (antibiotics + mechanical debridement) demonstrated a significantly greater reduction in BOP compared to the control group (WMD = −12.45, 95% CI: [−24.13, −0.77], P = 0.04; heterogeneity:I^2^ = 94%, P < 0.00001; Hartung–Knapp–Sidik–Jonkman:MD = −12.46, P = 0.0445). Subgroup analysis revealed no significant difference between local (WMD = −18.23, 95% CI: [−37.41, 0.96], P = 0.06; heterogeneity:I^2^ = 96%, P < 0.00001) and systemic (WMD = −4.47, 95% CI: [−15.22, 6.27], P = 0.41; heterogeneity:I^2^ = 77%, P = 0.0007;) antibiotic administration(Supplementary Materials S5 Fig in S1 File). No statistically significant differences were observed across subgroups of different antibiotics(Supplementary Materials S11 Fig in S1 File). Subgroup analyses stratified by follow-up duration showed no statistically significant differences across different follow-up time points(Supplementary Materials S7 Fig in S1 File).

### Plaque score (PS)

**Fig 4:**
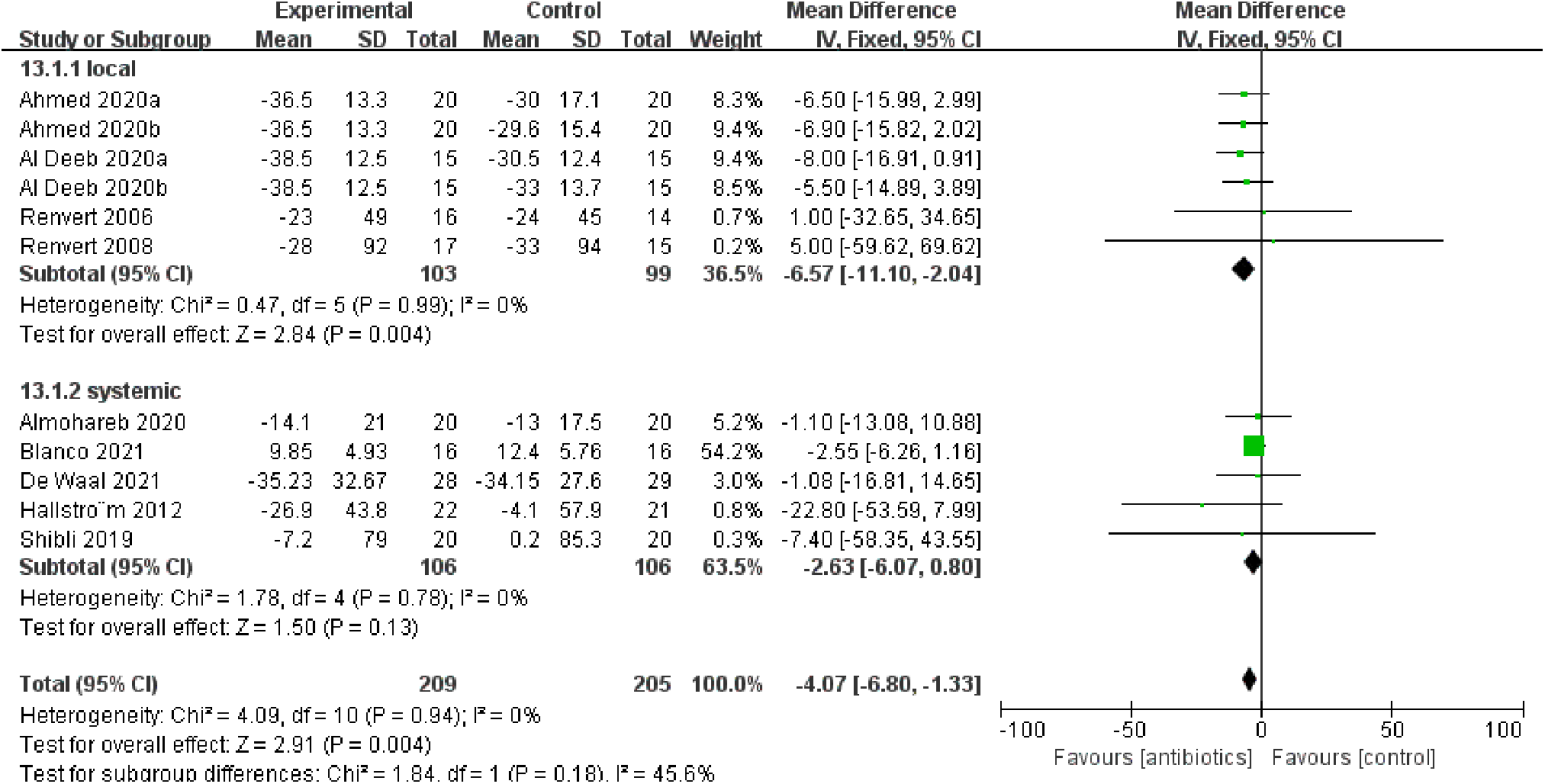
Forest plot of plaque score (PS). a and b are different groups of the same trial. local: local antibiotics；systemic：systemic antibiotics. Experimental group: antibiotics plus mechanical debridement. Control group: mechanical debridement; mechanical debridement plus enamel matrix derivative; mechanical debridement plus antimicrobial photodynamic therapy; mechanical debridement plus probiotic. NOTE:PS: mean difference in percentage of positive sites (%).

Nine trials reported PS outcomes. Fig 4 indicates that the experimental group exhibited a marked reduction in plaque scores compared to controls (WMD = −4.07, 95% CI: [−6.80, −1.33], P = 0.004; heterogeneity:I^2^ = 0%, P = 0.94). Route-specific analysis showed that local antibiotics significantly lowered PS (WMD = −6.57, 95% CI: [−11.10, −2.04], P = 0.004; heterogeneity:I^2^ = 0%, P = 0.99), whereas systemic administration did not reach statistical significance (WMD = −2.63, 95% CI: [−6.07, 0.80], P = 0.13; heterogeneity:I^2^ = 0%, P = 0.78)(Supplementary Materials S8 Fig in S1 File). Moreover, antibiotics as an adjunct to mechanical debridement proved superior to debridement alone. Subgroup analyses stratified by antibiotic type indicated a statistically significant difference in the amoxicillin group, while no significant differences were observed in the remaining subgroups(Supplementary Materials S14 Fig in S1 File). Subgroup analyses stratified by follow-up duration demonstrated a statistically significant difference only at the 6-month follow-up(Supplementary Materials S20 Fig in S1 File).

### Clinical attachment level (CAL)

**Fig 5:**
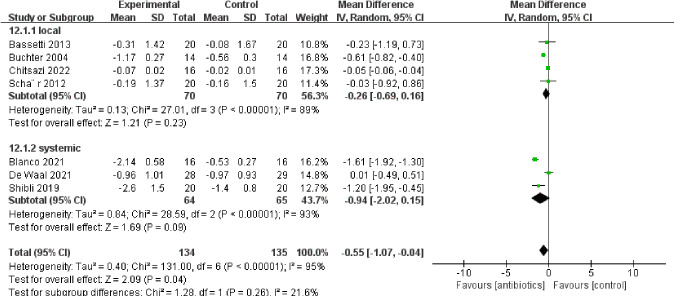
Forest plot of clinical attachment level (CAL). local: local antibiotics；systemic: systemic antibiotics. Experimental group: antibiotics plus mechanical debridement. Control group: mechanical debridement; mechanical debridement plus enamel matrix derivative; mechanical debridement plus antimicrobial photodynamic therapy; mechanical debridement plus probiotic. NOTE:CAL: mean difference in mm.

Seven trials evaluated CAL changes. As depicted in Fig 5, the experimental group demonstrated significantly greater CAL improvement than the control group (WMD = −0.55, 95% CI: [−1.07, −0.04], P = 0.04; heterogeneity:I^2^ = 95%, P < 0.00001; Hartung–Knapp–Sidik–Jonkman:MD = −0.552, P = 0.0675). Subgroup analysis(Supplementary Materials S7 Fig in S1 File) by administration route revealed comparable effects between local (WMD = −0.26, 95% CI: [−0.69, 0.16], P = 0.23; heterogeneity:I^2^ = 89%, P < 0.00001) and systemic (WMD = −0.94, 95% CI: [−2.02, 0.15], P = 0.09; heterogeneity:I^2^ = 93%, P < 0.00001) antibiotics. Importantly, the combination of antibiotics with mechanical debridement outperformed debridement alone. Subgroup analyses stratified by antibiotic type showed significant differences in subgroups containing only one study, while no statistically significant differences were detected in subgroups with two or more studies(Supplementary Materials S13 Fig in S1 File). Subgroup analyses based on follow-up duration revealed statistically significant differences at the 6-month and 12-month follow-up time points(Supplementary Materials S19 Fig in S1 File).

### Modified plaque index (mPLI)

Seven trials evaluated changes in mPLI. No statistically significant difference was observed between the experimental (antibiotics + mechanical debridement) and control groups (WMD = −0.07, 95% CI: [−0.16, 0.01], P = 0.09; heterogeneity:I^2^ = 26%, P = 0.2). Subgroup analysis by administration route revealed that local antibiotics did not significantly affect mPLI (WMD = −0.05, 95% CI: [−0.13, 0.04], P = 0.31; heterogeneity:I^2^ = 0%, P = 0.56), whereas systemic administration yielded a statistically significant reduction (WMD = −0.39, 95% CI: [−0.68, −0.09], P = 0.01; heterogeneity:I^2^ = 43%, P = 0.19). Detailed results are presented in the Supplementary Materials(S3 Fig, S10 Fig in S1 File). No statistically significant differences were observed across subgroups of different antibiotics(Supplementary Materials S16 Fig in S1 File). No significant differences were detected among subgroups with varying follow-up durations(Supplementary Materials S22 Fig in S1 File).

### Bone level (BL)

Seven trials investigated changes in BL. No statistically significant difference was detected between the experimental (antibiotics + mechanical debridement) and control groups regarding bone level (WMD = 0.03, 95% CI: [−0.04, 0.11], P = 0.39; heterogeneity:I^2^ = 0%, P = 0.45). Subgroup analyses further indicated no significant difference between local (WMD = 0.06, 95% CI: [−0.10, 0.22], P = 0.46; heterogeneity:I^2^ = 43%, P = 0.13) and systemic (WMD = 0.03, 95% CI: [−0.06, 0.11], P = 0.57; heterogeneity:I^2^ = 0%, P = 0.9) antibiotic administration. These findings are detailed in the Supplementary Materials S4 Fig and S9 Fig in S1 File. No statistically significant differences were observed across subgroups of different antibiotics(Supplementary Materials S15 Fig in S1 File). No significant differences were detected among subgroups with varying follow-up durations(Supplementary Materials S21 Fig in S1 File).

### Univariate meta-regression analyses

Univariate meta-regression analyses were performed for both PPD and BOP outcomes to explore potential sources of between-study heterogeneity, with prespecified covariates including study region, mean participant age, follow-up duration, proportion of male participants, publication year, and total sample size. For PPD, none of the covariates significantly explained the observed heterogeneity (all p > 0.05), with all R² values for explained heterogeneity being 0.00%. Similarly, for BOP, no significant association was found between any covariate and the magnitude of improvement (all p > 0.05); study region accounted for the highest proportion of explained heterogeneity (R² = 42.03%), while other covariates explained only a small proportion of the variability, ranging from 0.00% to 5.47%. Substantial residual heterogeneity remained in all adjusted models for both outcomes (I² > 89%, p < 0.0001), indicating that these geographic, demographic, and study design factors were not the main drivers of the observed heterogeneity in PPD and BOP reduction(Supplementary Materials S23-S34 Fig in S1 File).

### Sensitivity Analyses

A sensitivity analysis was conducted on probing pocket depth. The aggregated results remained unchanged following the sequential exclusion of each trial. Sensitivity analysis indicated that the results were stable. See the Supplementary Materials S35-S37 Fig in S1 File for relevant pictures.

### Publication Bias

Publication bias was evaluated through the analysis of a funnel plot related to probing pocket depth. The bilateral symmetric funnel plots demonstrated no significant evidence of publication bias(Egger ‘s test; P = 0.62) (Supplementary Materials S38-S40 Fig in S1 File).

## Discussion

Peri-implantitis is a primary cause of implant failure, and its treatment fundamentally aims to eliminate the biofilm from the implant surface, control inflammation, and thereby prevent further destruction of peri-implant soft and hard tissues [5]. While mechanical debridement serves as the cornerstone of clinical management, it alone often fails to completely remove biofilm embedded within implant threads and root surface concavities, leading to suboptimal outcomes and recurrent inflammation in some patients [9,23]. Consequently, the investigation of safe and effective adjunctive therapies has become a key research focus in implant dentistry. This study evaluated the efficacy of adjunctive antibiotics with mechanical debridement by assessing six core clinical parameters: bone level (BL), bleeding on probing (BOP), probing pocket depth (PPD), clinical attachment level (CAL), modified plaque index (mPLI), and plaque score (PS). Furthermore, four pre-specified subgroup analyses were conducted to elucidate potential differences in efficacy: (I) mechanical debridement plus antibiotics versus mechanical debridement alone, (II) local versus systemic antibiotic administration,(III) follow-up time and (IV) different antibiotics.

In terms of overall efficacy, the addition of antibiotics to mechanical debridement (the experimental group) demonstrated statistically significant improvements in soft tissue parameters and plaque control compared to control interventions (mechanical debridement alone, mechanical debridement with antimicrobial photodynamic therapy, or mechanical debridement with probiotics). The pooled effect size for probing pocket depth (PPD) (WMD = −0.69, 95% CI: [−1.04, −0.33], P = 0.0001) indicated a significantly greater reduction in the experimental group, consistent with recent systematic reviews showing that adjunctive antimicrobials can subgingival niches inaccessible to instruments and thereby reduce pocket depth [24,25]. For clinical attachment level (CAL), the pooled effect size (WMD = −0.55, 95% CI: [−1.07, −0.04], P = 0.04) suggested that adjunctive antibiotics effectively preserved clinical attachment and mitigated soft tissue recession, in line with evidence that systemic antibiotic regimens can yield additional CAL gain when combined with debridement [17,25]. Importantly, while this pooled weighted mean difference of 0.55 mm is relatively modest, it carries meaningful clinical implications in the context of non-surgical peri-implantitis treatment. In periodontology and implant dentistry, improvements in CAL of ≥0.5 mm are generally considered clinically relevant [26–28], as they reflect stabilization of the peri-implant attachment apparatus and reduced risk of further soft tissue recession and progressive bone loss. The lower bound of the 95% confidence interval (−1.07 mm) indicates that the true treatment effect could be as large as 1.07 mm, a magnitude widely recognized as clinically meaningful in this setting, and comparable to the CAL gains reported in previous systematic reviews of adjunctive antimicrobial therapies for peri-implantitis. Furthermore, the observed effect should be interpreted in light of the high between-study heterogeneity (I² = 95%) and the subgroup analysis showing a more pronounced, albeit non-significant, CAL benefit in the systemic antibiotic subgroup (WMD = −0.94 mm, 95% CI: −2.02 to 0.15), suggesting that the overall pooled estimate may mask differential efficacy according to the route of administration. Even modest CAL stabilization or improvement may reduce the risk of disease progression, the need for subsequent surgical intervention, and ultimately improve long-term implant survival—patient-centered outcomes of primary clinical importance. The pooled effect size for plaque score (PS) (WMD = −4.07, 95% CI: [−6.80, −1.33], P = 0.004) confirmed the efficacy of the experimental group in controlling plaque accumulation and addressing the source of infection. For bleeding on probing (BOP), the pooled effect size (WMD = −12.45, 95% CI: [−24.13, −0.77], P = 0.04) indicated a significant reduction in peri-implant tissue inflammation, echoing findings that local antibiotic delivery can significantly decrease bleeding scores with minimal adverse effects [29,30]. However, no statistically significant differences were observed for bone level (BL; pooled effect size: 0.03; 95% CI: [−0.04, 0.11]; P = 0.39) or modified plaque index (mPLI; pooled effect size: −0.07; 95% CI: [−0.16, 0.01]; P = 0.09). The lack of significant improvement in BL is not unexpected; as previous longitudinal and systematic reviews have noted, the re-establishment of bone fill following peri-implantitis treatment requires a prolonged observation period, and short-term follow-ups in the included RCTs may be insufficient to capture radiographic bone gain, despite clinical improvements in soft tissue parameters [31]. Considering the extremely high inter-study heterogeneity detected for PPD and BOP, univariate meta-regression analyses were performed to explore potential sources of heterogeneity, with study region, participant age, male proportion, follow-up duration, publication year and sample size included as covariates. The results demonstrated that none of the above demographic, geographical and study design-related factors could effectively account for the heterogeneity of the two indicators. Only study region partially explained the heterogeneity of BOP, with an R² of 42.03%, though this association did not reach statistical significance. Extremely high residual heterogeneity remained in all models after adjustment for covariates. These findings indicated that geographical variations and basic demographic characteristics of participants were not the main contributors to the inconsistent treatment effects across included studies. The observed heterogeneity was more likely attributed to unmeasured confounding factors, such as different intervention protocols, baseline severity of peri-implant lesions, smoking status, systemic comorbidities and compliance with periodontal maintenance.

Subgroup analyses further elucidated the differential efficacy associated with distinct treatment regimens, routes of administration, and temporal dynamics.

In the comparison of mechanical debridement plus antibiotics versus mechanical debridement alone, adjunctive antibiotics were associated with significant improvements in CAL (pooled effect size: −0.84; 95% CI: [−1.55, −0.13]; P = 0.02), PPD (−0.73; [−1.19, −0.27]; P = 0.002) and PS (−3.94; [−7.14, −0.74]; P = 0.02), while the reduction in BOP approached significance (−18.64; [−38.91, 1.63]; P = 0.07). These findings suggest a modest benefit of adjunctive antibiotics beyond that achieved by debridement alone.

When comparing local versus systemic antibiotic administration, comparable efficacy in reducing PPD was observed (local: −0.68; [−1.14, −0.22], P = 0.004; systemic: −0.70; [−1.25, −0.15], P = 0.01), with no significant difference between subgroups (P = 0.95, I² = 0%). For mPLI, systemic antibiotics showed a statistically significant improvement (−0.39; [−0.68, −0.09], P = 0.01), whereas local antibiotics did not (−0.05; [−0.13, 0.04], P = 0.31). In terms of PS, however, local delivery produced a significantly greater improvement (−6.57; [−11.10, −2.04], P = 0.004) compared to systemic administration (−2.63; [−6.07, 0.80], P = 0.13). This targeted efficacy of local application can be attributed to sustained high drug concentrations at the defect site, which effectively eradicate biofilm pathogens while minimizing systemic exposure and adverse effects [29,30]. Given the clinical safety concerns associated with systemic antibiotics—particularly the risks of gut dysbiosis and the emergence and spread of antibiotic resistance—local drug delivery remains the preferred strategy for managing peri‑implant infections [32].

The antibiotics used in the included studies were predominantly those commonly employed in dentistry, including amoxicillin, azithromycin, metronidazole, tetracycline, doxycycline, and minocycline, with metronidazole and tetracyclines being the most frequent. This pattern mirrors the anaerobic Gram‑negative microbiology of peri‑implantitis, against which these agents exhibit potent activity [33,34]. Notably, the overall effect sizes indicate that adjunctive antibiotics confer clinical benefits irrespective of the specific agent chosen from among these commonly used options, supporting flexibility in individualized treatment selection based on antimicrobial susceptibility, disease severity, and patient systemic conditions. Moreover, the included trials recruited patients from 12 countries across Asia, Europe, Oceania, and the Middle East, which substantially enhances the generalizability of the findings. Importantly, despite considerable heterogeneity (e.g., I² = 94% for BOP and 91% for PPD in the overall analyses), the consistent direction of treatment effects underscores the robustness of adjunctive antibiotic therapy when combined with mechanical debridement.

To further capture time‑dependent efficacy, we performed subgroup analyses stratified by follow‑up duration. These analyses revealed that the benefits of antibiotics were predominantly concentrated on soft tissue inflammation (BOP, PS), probing pocket depth (PPD), and clinical attachment level (CAL), with no significant improvements observed in plaque control (mPLI) or bone loss (BL). The temporal trends can be characterized as follows: (i) at 3 months (short‑term), beneficial trends or significant improvements were seen in BOP, PS, PPD, and mPLI, reflecting the rapid anti‑inflammatory effects of antibiotics on acute infection; (ii) at 6 months (mid‑term), the benefits for BOP and mPLI attenuated, while improvements in CAL and PS began to emerge or strengthen, suggesting a delayed effect on attachment gain; and (iii) at 12 months (long‑term), CAL benefits increased significantly and PPD benefits reappeared, whereas BOP and PS effects diminished, indicating that the long‑term value of antibiotics lies more in stabilizing attachment levels and probing depths rather than in controlling inflammation. In conclusion, the clinical role of adjunctive antibiotics in peri‑implantitis should be redefined: their primary functions are short‑term control of acute inflammation, mid‑term stabilization of attachment levels, and long‑term attenuation of probing depth progression, rather than reversal of bone loss or replacement of mechanical plaque control. In clinical practice, mechanical non‑surgical therapy should remain the cornerstone of treatment, and antibiotics should be reserved as adjuncts during acute inflammatory episodes to avoid over‑reliance on antimicrobial therapy.

### Advantages

First, the search strategy was rigorous and comprehensive, and the methodological quality of the included studies was high. A systematic search of five major international databases and clinical trial registries was conducted, with the search strategy strictly following PRISMA guidelines to minimize the risk of omitting relevant randomized controlled trials (RCTs). Stringent inclusion and exclusion criteria were applied, restricting inclusion to RCTs with control groups involving mechanical debridement alone, mechanical debridement plus antimicrobial photodynamic therapy, or mechanical debridement plus probiotics. The exclusion of non-randomized and retrospective studies effectively reduced selection bias and ensured the scientific rigor and reliability of the included evidence. Second, the included sample size was adequate and geographically representative. A total of 22 RCTs were included, representing a larger sample size and enhanced statistical power compared to previous meta-analyses. This allowed for more precise pooling of effect sizes and reduced the influence of individual study bias on the overall results. The inclusion of patients from 12 countries across diverse regions and ethnicities, with follow-up durations of 3, 6, and 12 months, and antibiotic types reflecting common clinical use, significantly enhances the generalizability and clinical applicability of the findings. Third, the outcome measures were comprehensive, and the subgroup analyses were highly targeted. This study incorporated six core clinical parameters encompassing hard tissues (BL), soft tissues (BOP, PPD, CAL), and plaque control (mPLI, PS), enabling a thorough and objective evaluation of treatment efficacy for peri-implantitis. Furthermore, four key pre-specified subgroup analyses—“mechanical debridement plus antibiotics versus mechanical debridement alone”, “local versus systemic antibiotic administration”, “different antibiotics” and “different follow-up time”—were conducted to precisely delineate differences in efficacy and the clinical scenarios best suited to each approach.

### Limitations

Several limitations of this systematic review and meta-analysis warrant careful consideration.

### Residual Heterogeneity and Unexplained Effect Modification

Although we systematically explored prespecified potential sources of heterogeneity—performing subgroup analyses based on the route of antibiotic administration (local vs. systemic), different antibiotic types, and different follow-up durations, as well as conducting meta-regression on variables including mean age, country, proportion of males, publication year, follow-up duration, and total sample size—substantial residual heterogeneity was still observed for the primary outcomes. This indicates that some important effect modifiers remain unidentified and unquantified. Due to insufficient information in the included trials, we were unable to further explore the contribution of the following known or potential determinants of treatment response to the heterogeneity: implant surface characteristics, initial severity of peri-implantitis, maintenance therapy protocols, patients’ oral hygiene status, smoking prevalence, systemic conditions such as diabetes, as well as the specific dosage and duration of antibiotic therapy. The lack of these data limits our ability to fully account for between-study variability through subgroup analyses or meta-regression, leaving the sources of residual heterogeneity incompletely elucidated. In addition, subtle differences across studies in the control interventions (mechanical debridement alone vs. mechanical debridement combined with other adjunctive therapies) and in the measurement and definition of outcome parameters may also constitute partial sources of heterogeneity, but could not be thoroughly quantified owing to incomplete reporting. Future studies should report the aforementioned patient- and treatment-related characteristics in greater detail to support more granular analyses of effect modification.

### Diagnostic Criteria Heterogeneity

Diagnostic criteria heterogeneity across included studies represents another important methodological consideration. While we adopted a standardized definition of peri-implantitis for study inclusion (bone loss ≥2 mm, probing pocket depth ≥5 mm, and presence of bleeding on probing) based on contemporary consensus guidelines [35], we acknowledge that diagnostic thresholds have evolved over time, and not all included trials applied identical, explicit criteria. Older studies, in particular, may have used less stringent definitions of peri-implantitis, relied on different baseline reference points for bone level measurements, or failed to report all components of the diagnostic triad in detail. This variability could introduce subtle clinical heterogeneity, which we attempted to account for through the use of a random-effects model and multiple subgroup analyses. Nonetheless, the consistent direction of treatment effects across studies supports the robustness of our main findings, despite these differences in diagnostic framing. Future trials would benefit from the universal adoption of standardized, consensus-based diagnostic criteria for peri-implantitis to enhance comparability and evidence synthesis.

### Incomplete Reporting and Confounding by Smoking Status

Smoking status is a well-established confounding factor in peri-implantitis treatment outcomes, yet its influence was not thoroughly examined in the present analysis owing to incomplete and inconsistent reporting. Although some of the included studies reported smoking status, the information was often fragmentary. Of the 22 studies reviewed, only 10 provided simple reporting of smoking status, while 8 studies used smoking only as an exclusion criterion without reporting baseline distribution, and 4 studies did not mention smoking at all. Moreover, among the studies that did report smoking, none performed subgroup analyses based on smoking status or explicitly tested whether smoking was balanced between treatment groups. This lack of adequate reporting and analysis limits our ability to assess the potential confounding effect of smoking on the observed treatment outcomes. It is possible that differential distributions of smokers across groups could have biased the results either toward or away from the null. Therefore, the findings of this meta-analysis should be interpreted with caution, and future randomized controlled trials should adhere to the CONSORT statement by clearly reporting smoking status at baseline (categorizing at least into current, former, and never smokers), quantifying smoking intensity (e.g., pack-years or cigarettes per day), and, when possible, conducting subgroup or sensitivity analyses stratified by smoking status to better elucidate its impact on therapeutic efficacy.

### Underreporting of Adverse Events and Antibiotic Resistance

A further limitation is the absence of a systematic analysis of adverse effects and antimicrobial resistance, which restricts the clinical applicability of our findings. Although the use of adjunctive systemic or local antibiotics may provide additional clinical benefits in selected cases of peri‑implantitis, the available evidence on adverse events remains heterogeneous and often under‑reported. Among the 22 randomised controlled trials included in this review, only six studies provided detailed information on adverse events, while the remaining studies either reported only descriptive data or did not mention adverse events at all. The reported adverse events were generally mild and transient, including gastrointestinal disturbances (nausea, diarrhoea, metallic taste) in 4–38% of patients receiving systemic amoxicillin, metronidazole or azithromycin, local pain or gingival soreness in up to 31% of patients after local minocycline delivery, and occasional cases of headache, dizziness or vaginal yeast infection. Importantly, no life‑threatening or irreversible adverse events were documented in any of the included trials.

More concerning is the almost complete lack of reporting on antibiotic resistance development. None of the included studies systematically monitored changes in the susceptibility profiles of peri‑implant pathogens before and after antibiotic therapy. This omission is critical, as repeated or widespread use of antibiotics—particularly metronidazole, amoxicillin and tetracyclines—in the context of peri‑implantitis treatment may contribute to the selection of resistant bacterial strains, not only at the treated site but also in the oral and gut microbiomes. The potential for gut dysbiosis following systemic antibiotic administration, briefly mentioned in our Discussion, warrants more attention. Furthermore, the microbiological outcomes reported in the included trials were inconsistent: while some studies showed a short‑term reduction in red‑complex pathogens (P. gingivalis, T. forsythia, T. denticola), others demonstrated rapid recolonisation within 3–6 months after treatment, and no study provided long‑term data on the emergence of resistant clones or on the ecological impact on the commensal oral microbiota.

In light of the growing emphasis on antibiotic stewardship in dentistry, the lack of robust safety and resistance data severely limits the ability to formulate evidence‑based recommendations on the routine use of adjunctive antibiotics for peri‑implantitis. Future trials should systematically collect and report adverse events using standardised tools (e.g., CTCAE or common toxicity criteria), include longitudinal monitoring of antimicrobial susceptibility (e.g., minimum inhibitory concentrations for key pathogens before and after therapy), and assess changes in the overall microbial community structure (metagenomic analysis) to capture both resistance emergence and potential dysbiosis. Until such data become available, clinicians should carefully weigh the modest and often transient clinical benefits of adjunctive antibiotics against the potential individual and public health risks, reserving their use for well‑defined cases (e.g., deep pockets with suppuration or immunocompromised patients) and ideally guided by microbiological diagnostics.

## Conclusion

Current evidence indicates that adjunctive systemic antibiotics combined with mechanical debridement may offer modest short-term improvements in soft tissue parameters and plaque control, including reductions in probing pocket depth (PPD), gains in clinical attachment level (CAL), and decreases in plaque score (PS) and bleeding on probing (BOP). However, the observed effect sizes are relatively small—particularly for PPD reduction—and no significant benefit has been demonstrated for radiographic bone level changes. These findings should be interpreted with caution due to substantial heterogeneity, variable follow-up durations, and the absence of long-term outcomes. Consequently, while adjunctive antibiotics could be considered in selected patients who respond inadequately to mechanical debridement alone, the current evidence does not support their routine widespread application in peri‑implantitis therapy. Further well-designed, long-term trials are needed to define the precise role of adjunctive antibiotics.

## Supporting information

S1 File Supplementary Materials. (ZIP)
