# Supplementary material for: Efficacy of adjunctive antibiotics compared to non-antibiotic therapies following mechanical debridement for peri-implantitis: A systematic review and meta-analysis of randomized controlled trials
Source: PLoS One. 2026 Jun 25;21(6):e0352311. doi: 10.1371/journal.pone.0352311 (PMC13298766; doi:10.1371/journal.pone.0352311)

**S1 Table：Methods:** Search strategy

**S1 Fig.** Summary of risks of bias of all included studies

**S2 Fig.** The GRADE Rating in all included studies

**S3 Fig.** Forest plot of modified plaque index (mPLI)

**S4 Fig.** Forest plot of Bone level (BL)

**S5 Fig.** Forest plot of comparing mechanical debridement plus antibiotics with mechanical debridement in bleeding on probing (BOP)

**S6 Fig.** Forest plot of comparing mechanical debridement plus antibiotics with mechanical debridement in probing pocket depth (PPD)

**S7 Fig.** Forest plot of comparing mechanical debridement plus antibiotics with mechanical debridement in clinical attachment level (CAL)

**S8 Fig.** Forest plot of comparing mechanical debridement plus antibiotics with mechanical debridement in plaque score (PS)

**S9 Fig.** Forest plot of comparing mechanical debridement plus antibiotics with mechanical debridement in bone level (BL)

**S10 Fig.** Forest plot of comparing mechanical debridement plus antibiotics with mechanical debridement in modified plaque index (mPLI)

**S11 Fig.** Forest plot of different antibiotics in bleeding on probing (BOP)

**S12 Fig.** Forest plot of different antibiotics in probing pocket depth (PPD)

**S13 Fig.** Forest plot of different antibiotics in clinical attachment level (CAL)

**S14 Fig.** Forest plot of different antibiotics in plaque score (PS)

**S15 Fig.** Forest plot of different antibiotics in bone level (BL)

**S16 Fig.** Forest plot of different antibiotics in modified plaque index (mPLI)

**S17 Fig.** Forest plot of follow-up time in bleeding on probing (BOP)

**S18 Fig.** Forest plot of follow-up time in probing pocket depth (PPD)

**S19 Fig.** Forest plot of follow-up time in clinical attachment level (CAL)

**S20 Fig.** Forest plot of follow-up time in plaque score (PS)

**S21 Fig.** Forest plot of follow-up time in bone level (BL)

**S22 Fig.** Forest plot of follow-up time in modified plaque index (mPLI)

**S23 Fig.** Univariate meta- regression for BOP(age)

**S24 Fig.** Univariate meta- regression for BOP(country)

**S25 Fig.** Univariate meta- regression for BOP(follow-up time)

**S26 Fig.** Univariate meta- regression for BOP(male propotion)

**S27 Fig.** Univariate meta- regression for BOP(sample)

**S28 Fig.** Univariate meta- regression for BOP(publication year)

**S29 Fig.** Univariate meta- regression for PPD(age)

**S30 Fig.** Univariate meta- regression for PPD(country)

**S31 Fig.** Univariate meta- regression for PPD(follow-up time)

**S32 Fig.** Univariate meta- regression for PPD(male propotion)

**S33 Fig.** Univariate meta- regression for PPD(sample)

**S34 Fig.** Univariate meta- regression for PPD(publication year)

**S35 Fig.** Sensitivity analysis of probing pocket depth (PPD)

**S36 Fig.** Sensitivity analysis of bleeding on probing (BOP)

**S37 Fig.** Sensitivity analysis of clinical attachment level (CAL)

**S38 Fig.** Publication bias of probing pocket depth (PPD)

**S39 Fig.** Publication bias of bleeding on probing (BOP)

**S40 Fig.** Publication bias of plaque score (PS)

**NOTE:**

**PPD:** mean difference in mm

**BOP/PS:** mean difference in percentage of positive sites (%)

**CAL/BL:** mean difference in mm

**mPLI:** mean difference in index score

**S1 Table：Methods:** Search strategy

**For example:**

| PubMed | | |
| --- | --- | --- |
| No. | Query | Results |
| #1 | (((((((Peri-Implantitides[Title/Abstract]) OR (Peri Implantitis[Title/Abstract])) OR (Periimplantitis[Title/Abstract])) OR (Periimplantitides[Title/Abstract])) OR (peri-implantitis[Title/Abstract])) OR (peri-implant[Title/Abstract])) OR (periimplant[Title/Abstract])) OR (implant[Title/Abstract]) | 181441 |
| #2 | (((((((((((((((((((((((((((((((((((((((((((((((((((((((((((((((((((((((((((((((((((((((((((((((drug delivery[Title/Abstract]) OR (drug implants[Title/Abstract])) OR (drug compounding[Title/Abstract])) OR (drug release[Title/Abstract])) OR (local drug treatment[Title/Abstract])) OR (Medication[Title/Abstract])) OR (drug treatment[Title/Abstract])) OR (antibacterial agents[Title/Abstract])) OR (local drug administration[Title/Abstract])) OR (Antimicrobial[Title/Abstract])) OR (Bactericides[Title/Abstract])) OR (antiinfective agents[Title/Abstract])) OR (Antibiotics[Title/Abstract])) OR (systemic antibiotics[Title/Abstract])) OR (antibiotic prophylaxis[Title/Abstract])) OR (amoxicillin[Title/Abstract])) OR (Amoxil[Title/Abstract])) OR (Clamoxyl[Title/Abstract])) OR (Penamox[Title/Abstract])) OR (Wymox[Title/Abstract])) OR (Trimox[Title/Abstract])) OR (Polymox[Title/Abstract])) OR (Actimoxi[Title/Abstract])) OR (Hydroxyampicillin[Title/Abstract])) OR (doxycycline[Title/Abstract])) OR (Hydramycin[Title/Abstract])) OR (Doxycycline Calcium[Title/Abstract])) OR (Vibramycin[Title/Abstract])) OR (BMY28689[Title/Abstract])) OR (BU3839T[Title/Abstract])) OR (Vibravenos[Title/Abstract])) OR (Periostat[Title/Abstract])) OR (Atridox[Title/Abstract])) OR (Doryx[Title/Abstract])) OR (Oracea[Title/Abstract])) OR (minocycline[Title/Abstract])) OR (Minocin[Title/Abstract])) OR (Minox 50[Title/Abstract])) OR (Akamin[Title/Abstract])) OR (Akne Puren[Title/Abstract])) OR (Aknemin[Title/Abstract])) OR (Aknin Mino[Title/Abstract])) OR (Aknosan[Title/Abstract])) OR (Apo Minocycline[Title/Abstract])) OR (Arestin[Title/Abstract])) OR (Dynacin[Title/Abstract])) OR (Lederderm[Title/Abstract])) OR (Blemix[Title/Abstract])) OR (Cyclomin[Title/Abstract])) OR (Cyclops[Title/Abstract])) OR (Dentomycin[Title/Abstract])) OR (Icht Oral[Title/Abstract])) OR (Klinomycin[Title/Abstract])) OR (Minakne[Title/Abstract])) OR (Mino Wolff[Title/Abstract])) OR (Minoclir[Title/Abstract])) OR (Minolis[Title/Abstract])) OR (Minoplus[Title/Abstract])) OR (Mestacine[Title/Abstract])) OR (Minomycin[Title/Abstract])) OR (Minotab[Title/Abstract])) OR (Mynocine[Title/Abstract])) OR (metronidazole[Title/Abstract])) OR (Clont[Title/Abstract])) OR (Danizol[Title/Abstract])) OR (Flagyl[Title/Abstract])) OR (Gineflavir[Title/Abstract])) OR (Metric[Title/Abstract])) OR (MetroGel[Title/Abstract])) OR (Metrodzhil[Title/Abstract])) OR (Metrogyl[Title/Abstract])) OR (Satric[Title/Abstract])) OR (Trichazol[Title/Abstract])) OR (Trichopol[Title/Abstract])) OR (Trivazol[Title/Abstract])) OR (Vagilen[Title/Abstract])) OR (Bayer 5360[Title/Abstract])) OR (tetracycline[Title/Abstract])) OR (Topicycline[Title/Abstract])) OR (Achromycin V[Title/Abstract])) OR (Hostacyclin[Title/Abstract])) OR (Sustamycin[Title/Abstract])) OR (Tetrabid[Title/Abstract])) OR (4 Epitetracycline[Title/Abstract])) OR (Achromycin[Title/Abstract])) OR (Clindamycin[Title/Abstract])) OR (Chlorlincocin[Title/Abstract])) OR (Chlolincocin[Title/Abstract])) OR (Dalacin C[Title/Abstract])) OR (Cleocin[Title/Abstract])) OR (Chlorhexidine[Title/Abstract])) OR (Tubulicid[Title/Abstract])) OR (Sebidin A[Title/Abstract])) OR (Novalsan[Title/Abstract])) OR (MK412A[Title/Abstract])) | 1161739 |
| #3 | ((((((((Randomized Controlled Trial[Title/Abstract]) OR (randomized controlled trial[Title/Abstract])) OR (controlled clinical trial[Title/Abstract])) OR (randomized[Title/Abstract])) OR (placebo[Title/Abstract])) OR (clinical trials[Title/Abstract])) OR (randomly[Title/Abstract])) OR (trial[Title/Abstract])) OR (RCT[Title/Abstract]) | 2016702 |
| #4 | #1 AND #2 AND #3 | 1163 |

| Cochrane library | | |
| --- | --- | --- |
| No. | Query | Results |
| #1 | (Peri-Implantitides OR Peri Implantitis OR Periimplantitis OR Periimplantitides OR peri-implantitis OR peri-implant OR periimplant OR implant):ti,ab,kw | 18560 |
| #2 | (drug delivery OR drug implants OR drug compounding OR drug release OR local drug treatment OR Medication OR drug treatment OR antibacterial agents OR local drug administration OR Antimicrobial OR Bactericides OR antiinfective agents OR Antibiotics OR systemic antibiotics OR antibiotic prophylaxis OR amoxicillin OR Amoxil OR Clamoxyl OR Penamox OR Wymox OR Trimox OR Polymox OR Actimoxi OR Hydroxyampicillin OR doxycycline OR Hydramycin OR Doxycycline Calcium OR Vibramycin OR BMY28689 OR BU3839T OR Vibravenos OR Periostat OR Atridox OR Doryx OR Oracea OR minocycline OR Minocin OR Minox 50 OR Akamin OR Akne Puren OR Aknemin OR Aknin Mino OR Aknosan OR Apo Minocycline OR Arestin OR Dynacin OR Lederderm OR Blemix OR Cyclomin OR Cyclops OR Dentomycin OR Icht Oral OR Klinomycin OR Minakne OR Mino Wolff OR Minoclir OR Minolis OR Minoplus OR Mestacine OR Minomycin OR Minotab OR Mynocine OR metronidazole OR Clont OR Danizol OR Flagyl OR Gineflavir OR Metric OR MetroGel OR Metrodzhil OR Metrogyl OR Satric OR Trichazol OR Trichopol OR Trivazol OR Vagilen OR Bayer 5360 OR tetracycline OR Topicycline OR Achromycin V OR Hostacyclin OR Sustamycin OR Tetrabid OR 4 Epitetracycline OR Achromycin OR Clindamycin OR Chlorlincocin OR Chlolincocin OR Dalacin C OR Cleocin OR Chlorhexidine OR Tubulicid OR Sebidin A OR Novalsan OR MK412A):ti,ab,kw | 617646 |
| #3 | ("randomized controlled trial" OR "randomized controlled trial" OR "controlled clinical trial" OR "Randomized" OR "placebo" OR "clinical trials" OR "randomly" OR "Trial" OR "RCT"):ti,ab,kw | 1670441 |
| #4 | #1 AND #2 AND #3 | 3121 |

| Embase | | |
| --- | --- | --- |
| No. | Query | Results |
| #1 | 'Peri-Implantitides':ab,ti,kw OR 'Peri Implantitis':ab,ti,kw OR 'Periimplantitis':ab,ti,kw OR 'Periimplantitides':ab,ti,kw OR 'peri-implantitis':ab,ti,kw OR 'peri-implant':ab,ti,kw OR 'periimplant':ab,ti,kw OR 'implant':ab,ti,kw | 232393 |
| #2 | 'drug delivery':ab,ti,kw OR 'drug implants':ab,ti,kw OR 'drug compounding':ab,ti,kw OR 'drug release':ab,ti,kw OR 'local drug treatment':ab,ti,kw OR 'Medication':ab,ti,kw OR 'drug treatment':ab,ti,kw OR 'antibacterial agents':ab,ti,kw OR 'local drug administration':ab,ti,kw OR 'Antimicrobial':ab,ti,kw OR 'Bactericides':ab,ti,kw OR 'antiinfective agents':ab,ti,kw OR 'Antibiotics':ab,ti,kw OR 'systemic antibiotics':ab,ti,kw OR 'antibiotic prophylaxis':ab,ti,kw OR 'amoxicillin':ab,ti,kw OR 'Amoxil':ab,ti,kw OR 'Clamoxyl':ab,ti,kw OR 'Penamox':ab,ti,kw OR 'Wymox':ab,ti,kw OR 'Trimox':ab,ti,kw OR 'Polymox':ab,ti,kw OR 'Actimoxi':ab,ti,kw OR 'Hydroxyampicillin':ab,ti,kw OR 'doxycycline':ab,ti,kw OR 'Hydramycin':ab,ti,kw OR 'Doxycycline Calcium':ab,ti,kw OR 'Vibramycin':ab,ti,kw OR 'BMY28689':ab,ti,kw OR 'BU3839T':ab,ti,kw OR 'Vibravenos':ab,ti,kw OR 'Periostat':ab,ti,kw OR 'Atridox':ab,ti,kw OR 'Doryx':ab,ti,kw OR 'Oracea':ab,ti,kw OR 'minocycline':ab,ti,kw OR 'Minocin':ab,ti,kw OR 'Minox 50':ab,ti,kw OR 'Akamin':ab,ti,kw OR 'Akne Puren':ab,ti,kw OR 'Aknemin':ab,ti,kw OR 'Aknin Mino':ab,ti,kw OR 'Aknosan':ab,ti,kw OR 'Apo Minocycline':ab,ti,kw OR 'Arestin':ab,ti,kw OR 'Dynacin':ab,ti,kw OR 'Lederderm':ab,ti,kw OR 'Blemix':ab,ti,kw OR 'Cyclomin':ab,ti,kw OR 'Cyclops':ab,ti,kw OR 'Dentomycin':ab,ti,kw OR 'Icht Oral':ab,ti,kw OR 'Klinomycin':ab,ti,kw OR 'Minakne':ab,ti,kw OR 'Mino Wolff':ab,ti,kw OR 'Minoclir':ab,ti,kw OR 'Minolis':ab,ti,kw OR 'Minoplus':ab,ti,kw OR 'Mestacine':ab,ti,kw OR 'Minomycin':ab,ti,kw OR 'Minotab':ab,ti,kw OR 'Mynocine':ab,ti,kw OR 'metronidazole':ab,ti,kw OR 'Clont':ab,ti,kw OR 'Danizol':ab,ti,kw OR 'Flagyl':ab,ti,kw OR 'Gineflavir':ab,ti,kw OR 'Metric':ab,ti,kw OR 'MetroGel':ab,ti,kw OR 'Metrodzhil':ab,ti,kw OR 'Metrogyl':ab,ti,kw OR 'Satric':ab,ti,kw OR 'Trichazol':ab,ti,kw OR 'Trichopol':ab,ti,kw OR 'Trivazol':ab,ti,kw OR 'Vagilen':ab,ti,kw OR 'Bayer 5360':ab,ti,kw OR 'tetracycline':ab,ti,kw OR 'Topicycline':ab,ti,kw OR 'Achromycin V':ab,ti,kw OR 'Hostacyclin':ab,ti,kw OR 'Sustamycin':ab,ti,kw OR 'Tetrabid':ab,ti,kw OR '4 Epitetracycline':ab,ti,kw OR 'Achromycin':ab,ti,kw OR 'Clindamycin':ab,ti,kw OR 'Chlorlincocin':ab,ti,kw OR 'Chlolincocin':ab,ti,kw OR 'Dalacin C':ab,ti,kw OR 'Cleocin':ab,ti,kw OR 'Chlorhexidine':ab,ti,kw OR 'Tubulicid':ab,ti,kw OR 'Sebidin A':ab,ti,kw OR 'Novalsan':ab,ti,kw OR 'MK412A':ab,ti,kw | 1698366 |
| #3 | 'randomized controlled trial':ab,ti,kw OR 'controlled clinical trial':ab,ti,kw OR randomized:ab,ti,kw OR placebo:ab,ti,kw OR 'clinical trials':ab,ti,kw OR randomly:ab,ti,kw OR trial:ab,ti,kw OR RCT:ab,ti,kw | 3202368 |
| #4 | #1 AND #2 AND #3 | 2415 |

| Web of science | | |
| --- | --- | --- |
| No. | Query | Results |
| #1 | TI=(Peri-Implantitides OR Peri Implantitis OR Periimplantitis OR Periimplantitides OR peri-implantitis OR peri-implant OR periimplant OR implant) | 79402 |
| #2 | TI=(drug delivery OR drug implants OR drug compounding OR drug release OR local drug treatment OR Medication OR drug treatment OR antibacterial agents OR local drug administration OR Antimicrobial OR Bactericides OR antiinfective agents OR Antibiotics OR systemic antibiotics OR antibiotic prophylaxis OR amoxicillin OR Amoxil OR Clamoxyl OR Penamox OR Wymox OR Trimox OR Polymox OR Actimoxi OR Hydroxyampicillin OR doxycycline OR Hydramycin OR Doxycycline Calcium OR Vibramycin OR BMY28689 OR BU3839T OR Vibravenos OR Periostat OR Atridox OR Doryx OR Oracea OR minocycline OR Minocin OR Minox 50 OR Akamin OR Akne Puren OR Aknemin OR Aknin Mino OR Aknosan OR Apo Minocycline OR Arestin OR Dynacin OR Lederderm OR Blemix OR Cyclomin OR Cyclops OR Dentomycin OR Icht Oral OR Klinomycin OR Minakne OR Mino Wolff OR Minoclir OR Minolis OR Minoplus OR Mestacine OR Minomycin OR Minotab OR Mynocine OR metronidazole OR Clont OR Danizol OR Flagyl OR Gineflavir OR Metric OR MetroGel OR Metrodzhil OR Metrogyl OR Satric OR Trichazol OR Trichopol OR Trivazol OR Vagilen OR Bayer 5360 OR tetracycline OR Topicycline OR Achromycin V OR Hostacyclin OR Sustamycin OR Tetrabid OR 4 Epitetracycline OR Achromycin OR Clindamycin OR Chlorlincocin OR Chlolincocin OR Dalacin C OR Cleocin OR Chlorhexidine OR Tubulicid OR Sebidin A OR Novalsan OR MK412A) | 409168 |
| #3 | TI=(randomized controlled trial OR controlled clinical trial OR randomized OR placebo OR clinical trials OR randomly OR trial OR RCT) | 561131 |
| #4 | #1 AND #2 AND #3 | 104 |

| Scopus | | |
| --- | --- | --- |
| No. | Query | Results |
| #1 | TITLE-ABS-KEY(Peri-Implantitides OR Peri Implantitis OR Periimplantitis OR Periimplantitides OR peri-implantitis OR peri-implant OR periimplant OR implant) | 17179 |
| #2 | TITLE-ABS-KEY(drug delivery OR drug implants OR drug compounding OR drug release OR local drug treatment OR Medication OR drug treatment OR antibacterial agents OR local drug administration OR Antimicrobial OR Bactericides OR antiinfective agents OR Antibiotics OR systemic antibiotics OR antibiotic prophylaxis OR amoxicillin OR doxycycline OR minocycline OR tetracycline OR metronidazole OR Clindamycin OR Chlorhexidine) | 4225 |
| #3 | TITLE-ABS-KEY(randomized controlled trial OR controlled clinical trial OR randomized OR placebo OR clinical trials OR randomly OR trial OR RCT) | 962425 |
| #4 | #1 AND #2 AND #3 | 14 |

2026.1.15

clinicaltrials.gov:1167

Search Details: Viewing 1-10 out of 1,167 studies for: Peri-Implantitides OR Peri Implantitis OR Periimplantitis OR Periimplantitides OR peri-implantitis OR peri-implant OR periimplant OR implant | drug delivery OR drug implants OR drug compounding OR drug release OR local drug treatment OR Medication OR drug treatment OR antibacterial agents OR local drug administration OR Antimicrobial OR Bactericides OR antiinfective agents OR Antibiotics OR systemic antibiotics OR antibiotic prophylaxis OR amoxicillin OR doxycycline OR minocycline OR tetracycline OR metronidazole OR Clindamycin OR Chlorhexidine | Completed, Terminated studies

**S1 Fig.** Summary of risks of bias of all included studies


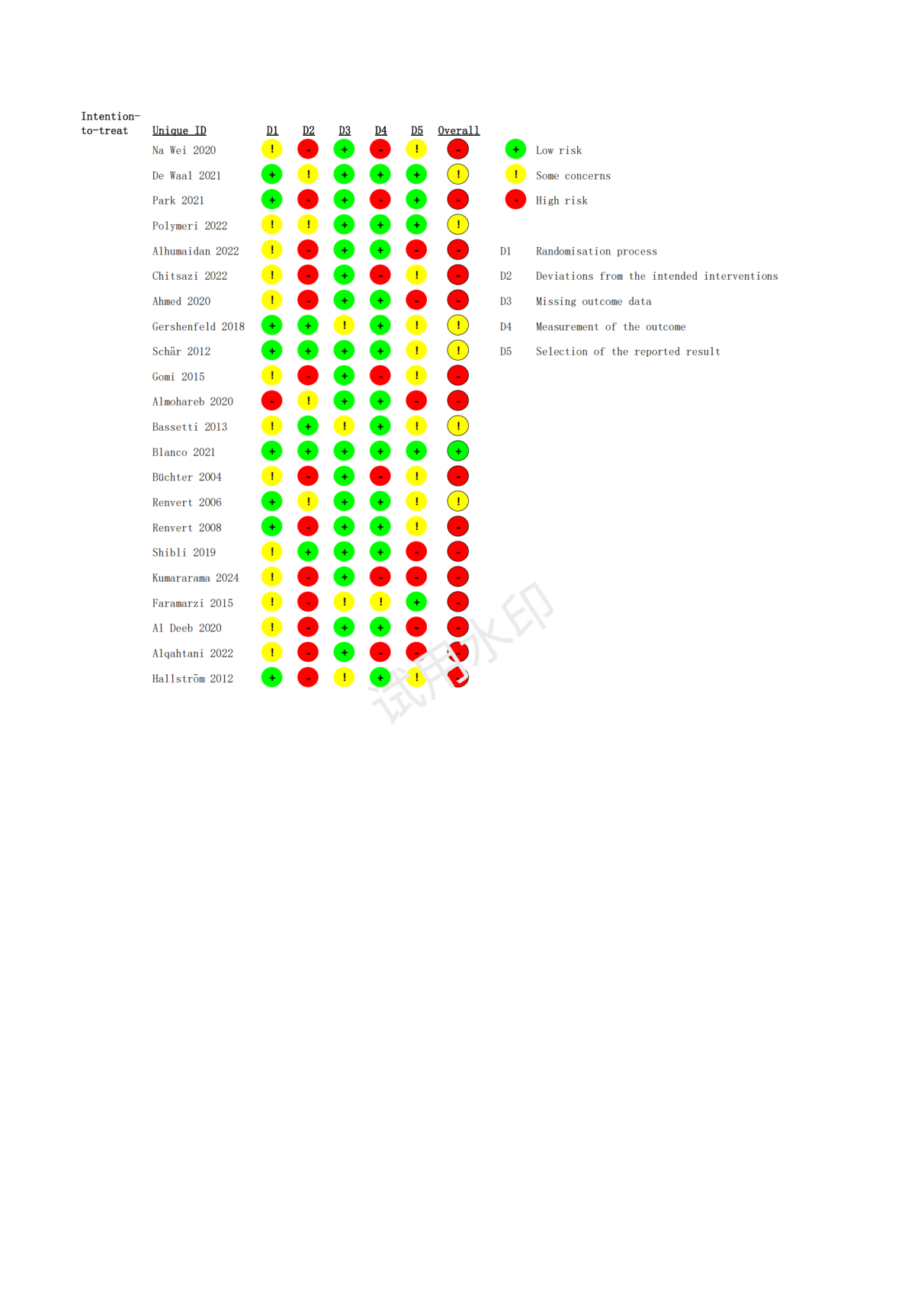


**S2 Fig.** The GRADE Rating in all included studies


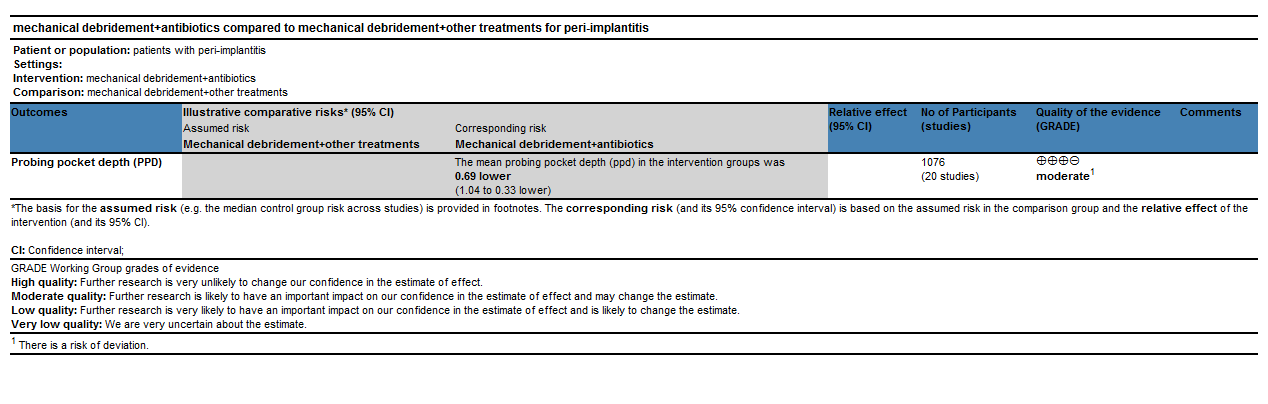


**S3 Fig.** Forest plot of modified plaque index (mPLI)


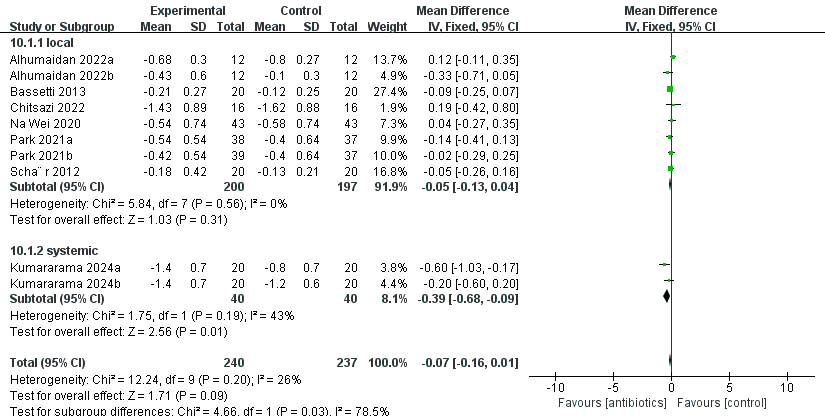


**S4 Fig.** Forest plot of Bone level (BL)


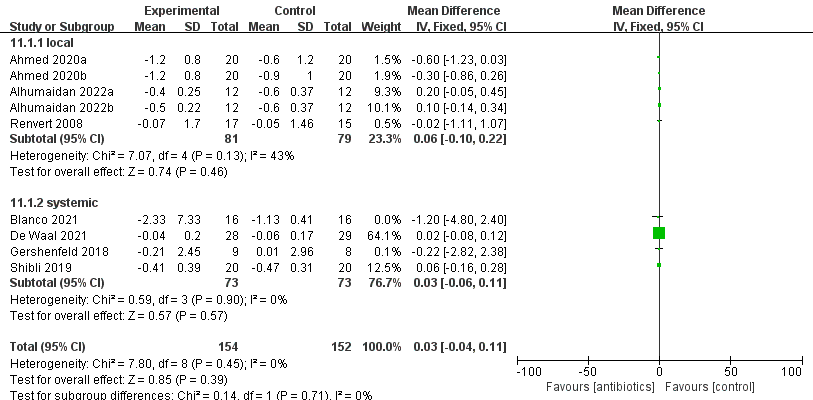


**S5 Fig.** Forest plot of comparing mechanical debridement plus antibiotics with mechanical debridement in bleeding on probing (BOP)


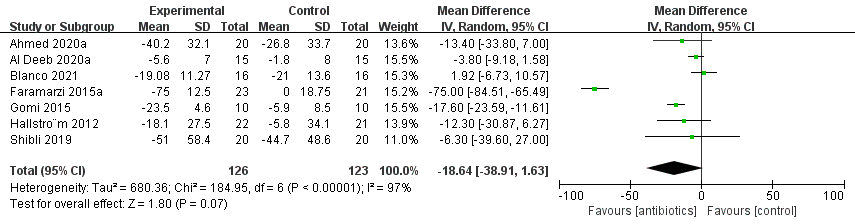


**S6 Fig.** Forest plot of comparing mechanical debridement plus antibiotics with mechanical debridement in probing pocket depth (PPD)


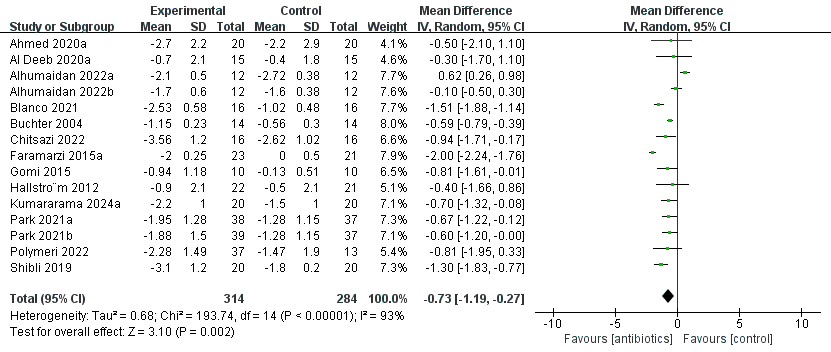


**S7 Fig.** Forest plot of comparing mechanical debridement plus antibiotics with mechanical debridement in clinical attachment level (CAL)
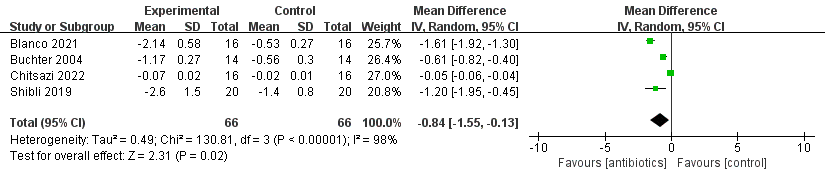


**S8 Fig.** Forest plot of comparing mechanical debridement plus antibiotics with mechanical debridement in plaque score (PS)


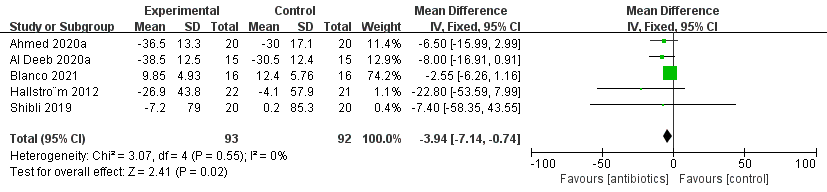


**S9 Fig.** Forest plot of comparing mechanical debridement plus antibiotics with mechanical debridement in bone level (BL)
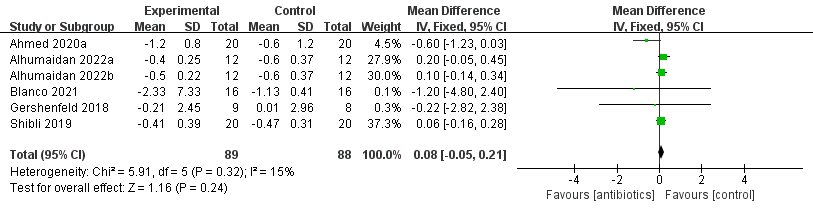


**S10 Fig.** Forest plot of comparing mechanical debridement plus antibiotics with mechanical debridement in modified plaque index (mPLI)
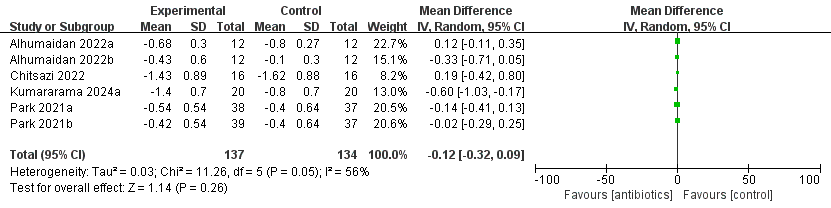


**S11 Fig.** Forest plot of different antibiotics in bleeding on probing (BOP)


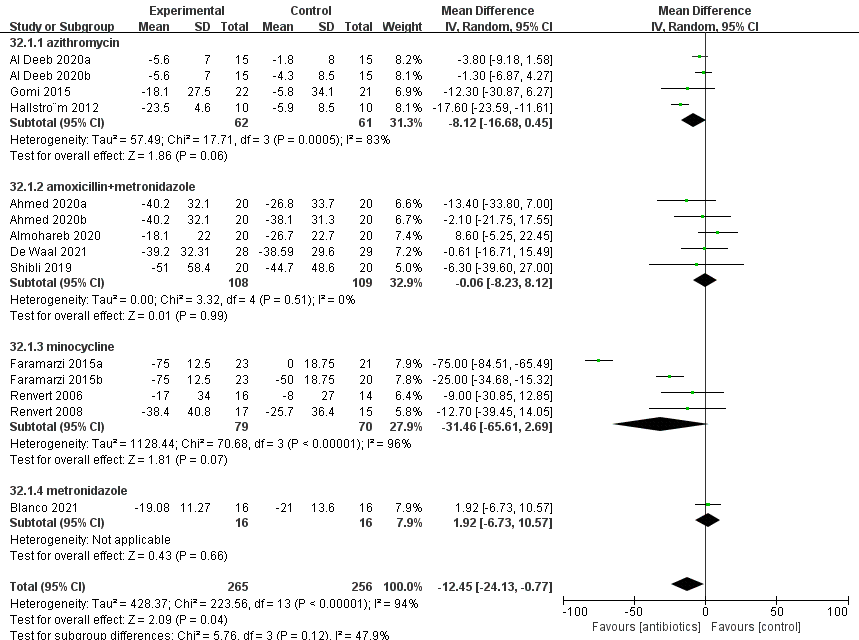


**S12 Fig.** Forest plot of different antibiotics in probing pocket depth (PPD)


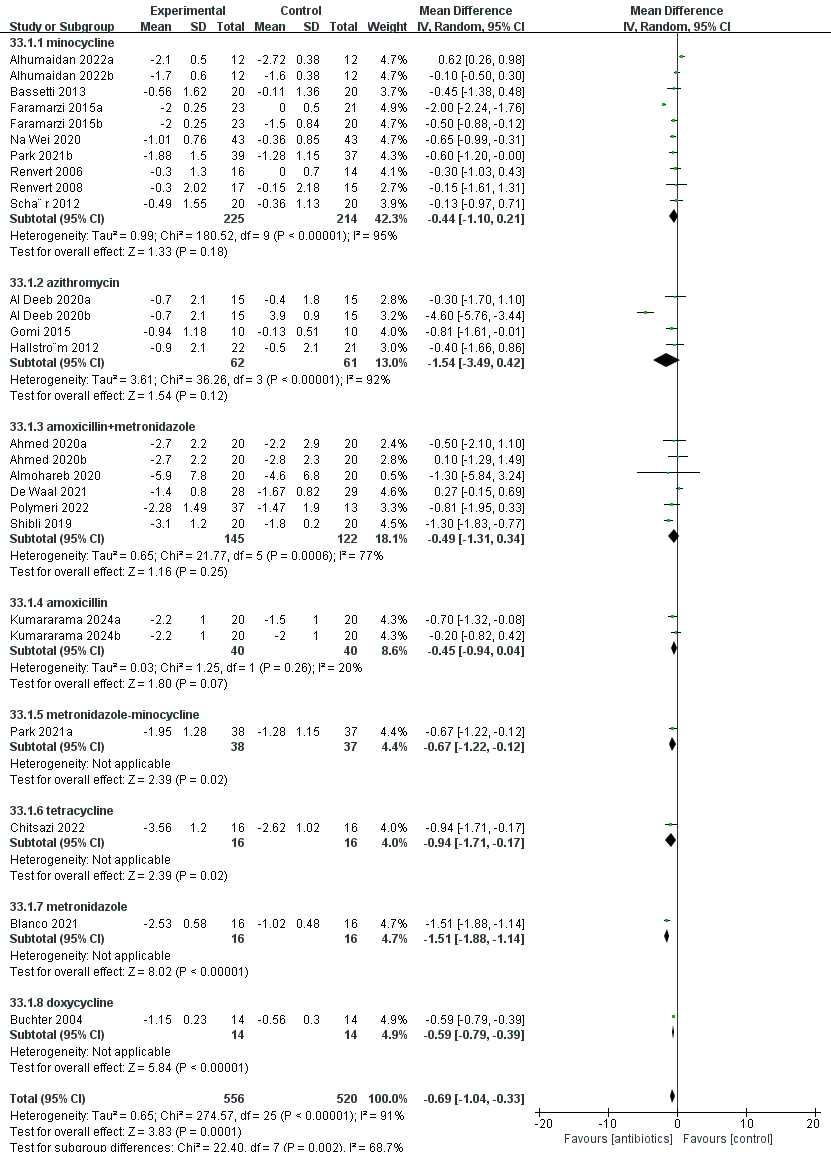


**S13 Fig.** Forest plot of different antibiotics in clinical attachment level (CAL)


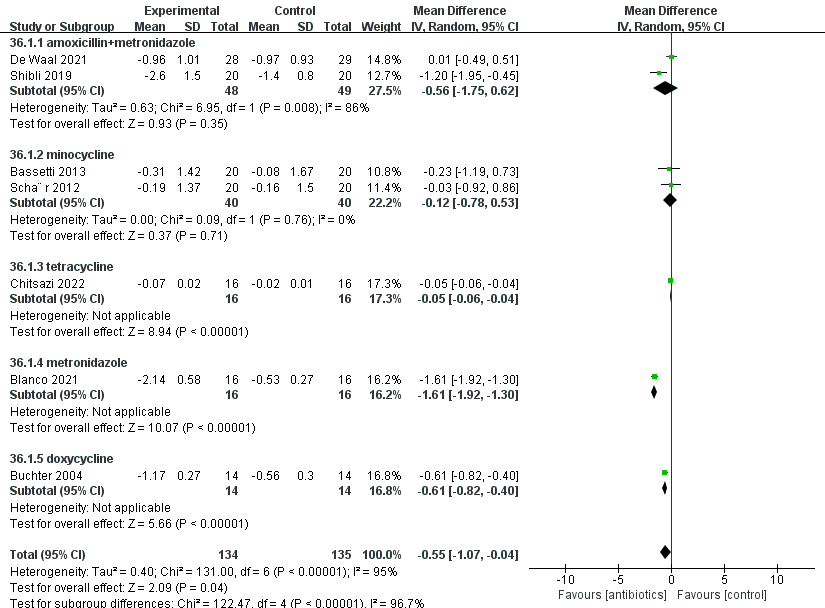


**S14 Fig.** Forest plot of different antibiotics in plaque score (PS)


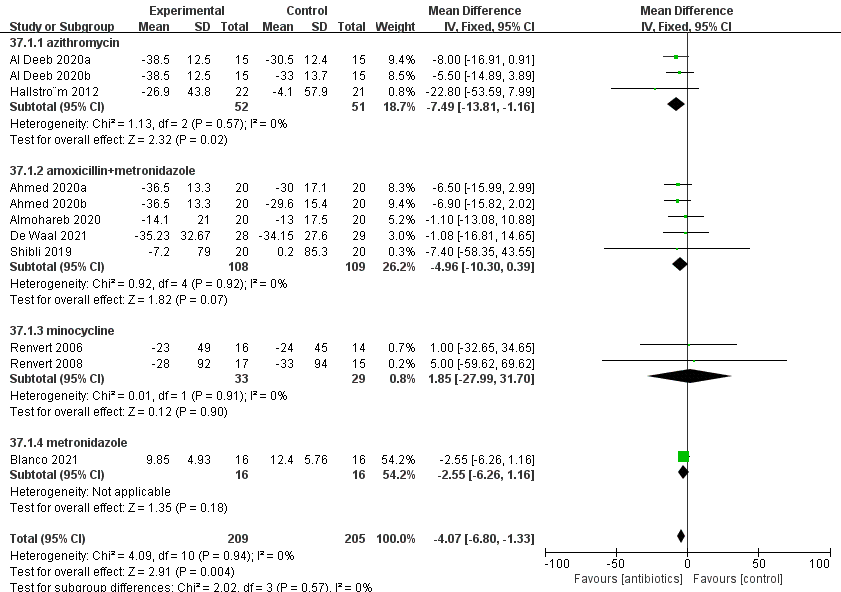


**S15 Fig.** Forest plot of different antibiotics in bone level (BL)


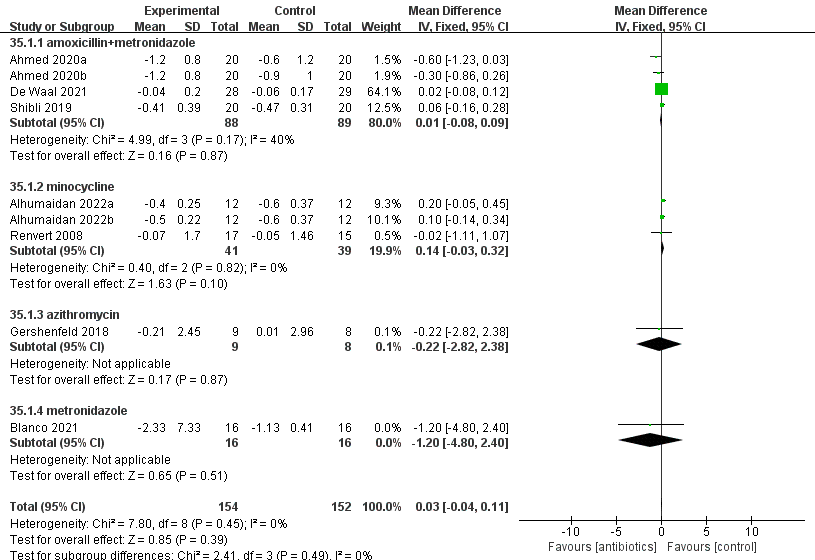


**S16 Fig.** Forest plot of different antibiotics in modified plaque index (mPLI)


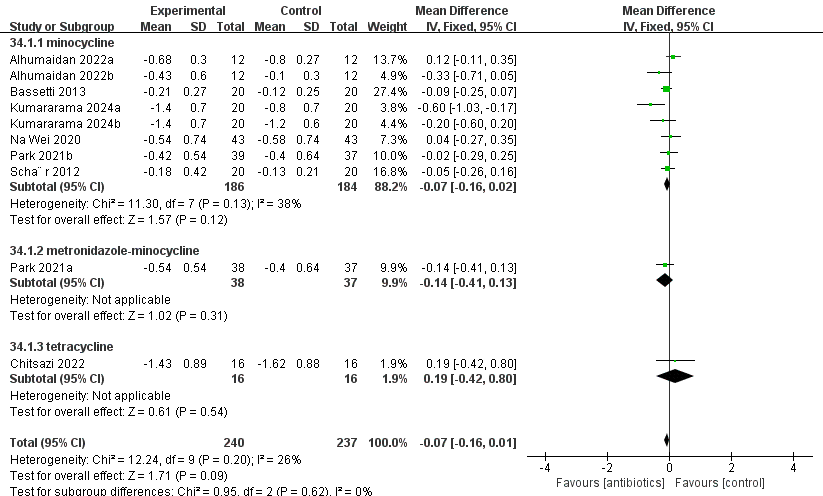


**S17 Fig.** Forest plot of follow-up time in bleeding on probing (BOP)


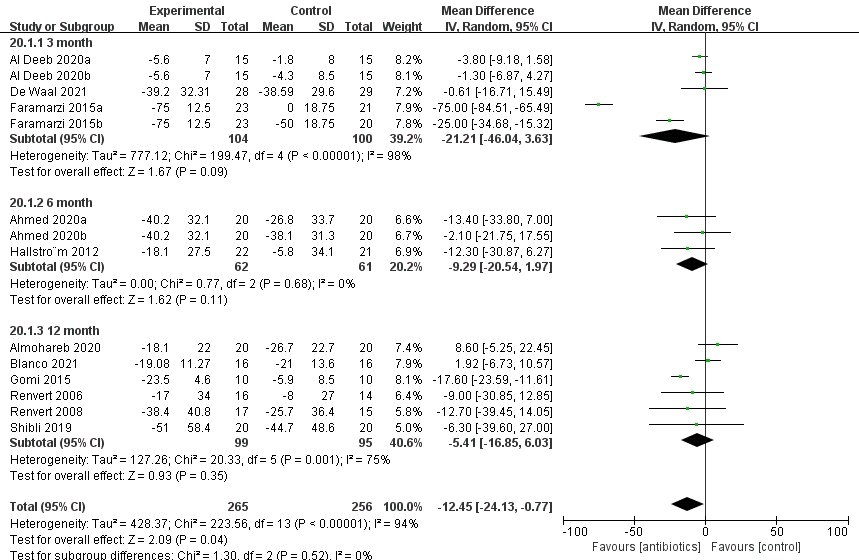


**S18 Fig.** Forest plot of follow-up time in probing pocket depth (PPD)


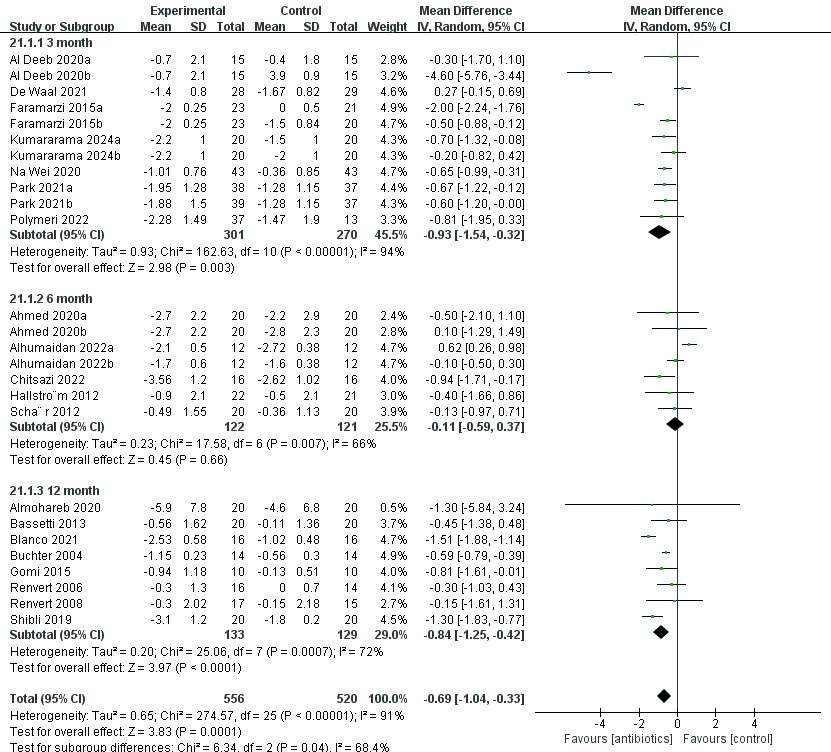


**S19 Fig.** Forest plot of follow-up time in clinical attachment level (CAL)


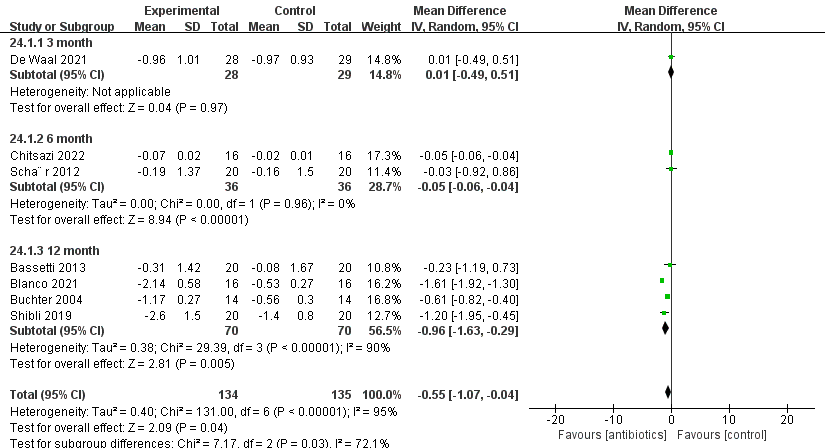


**S20 Fig.** Forest plot of follow-up time in plaque score (PS)


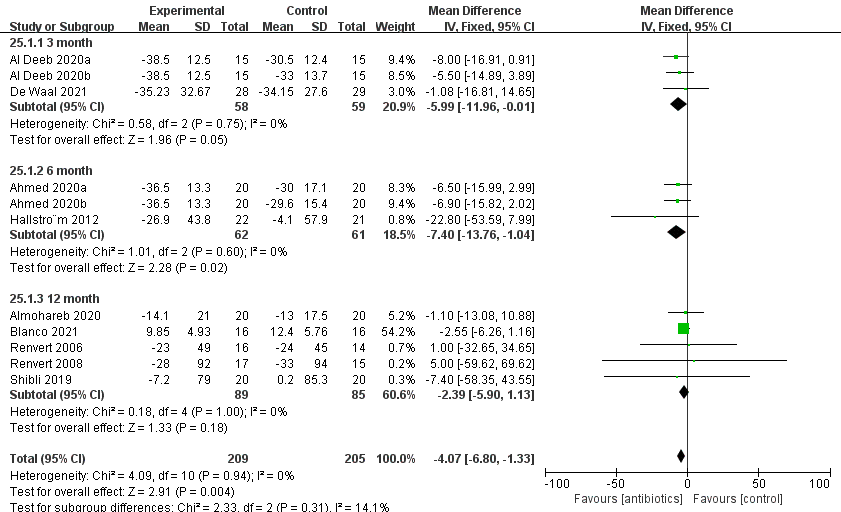


**S21 Fig.** Forest plot of follow-up time in bone level (BL)


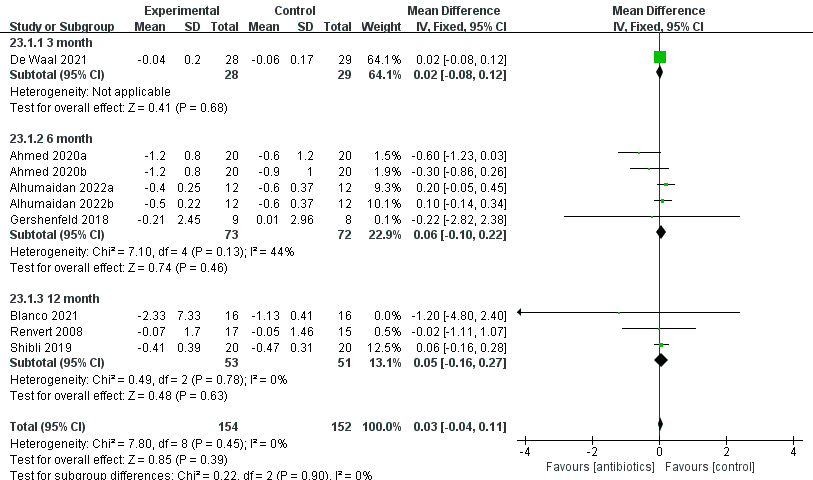


**S22 Fig.** Forest plot of follow-up time in modified plaque index (mPLI)


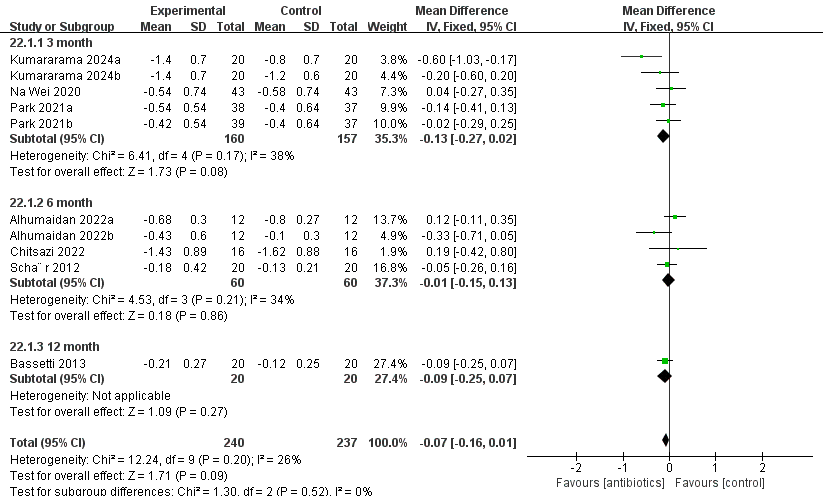


**S23 Fig.** Univariate meta- regression for BOP(age)


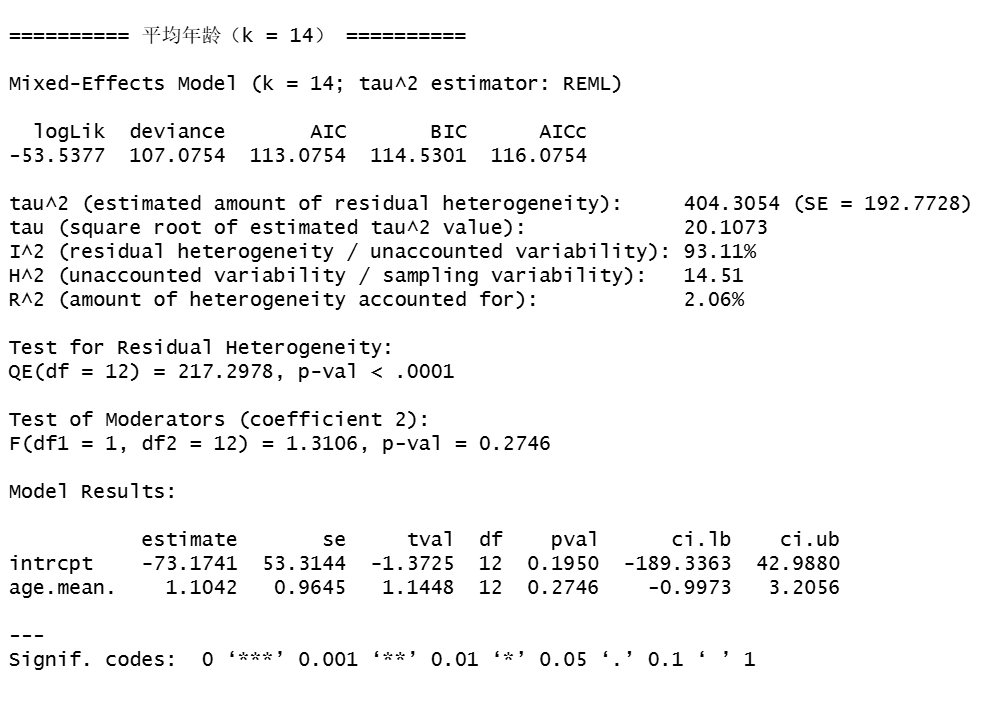


**S24 Fig.** Univariate meta- regression for BOP(country)


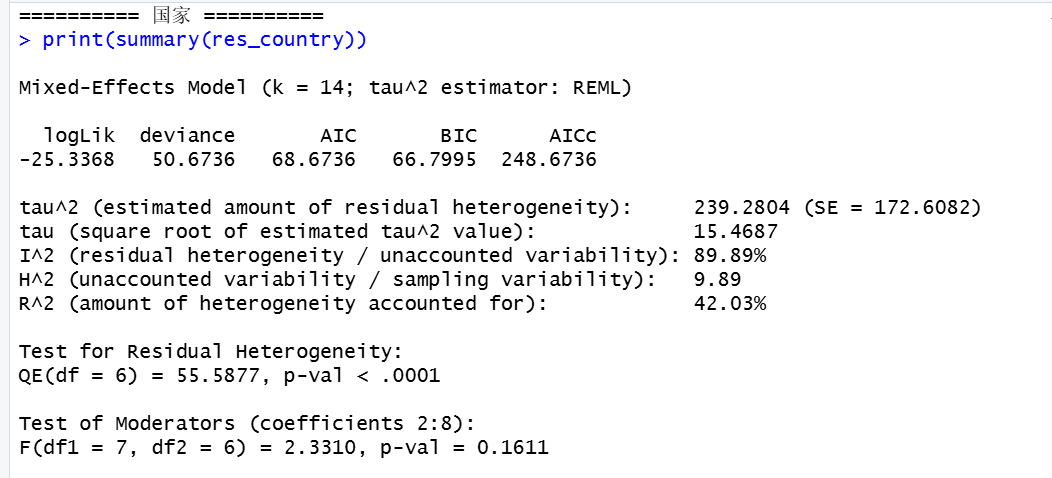


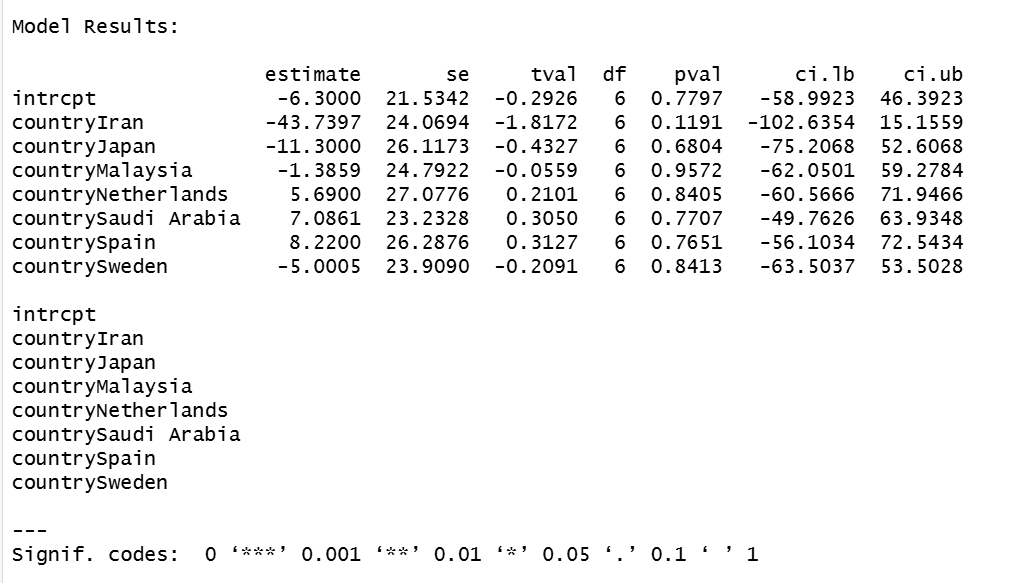


**S25 Fig.** Univariate meta- regression for BOP(follow-up time)


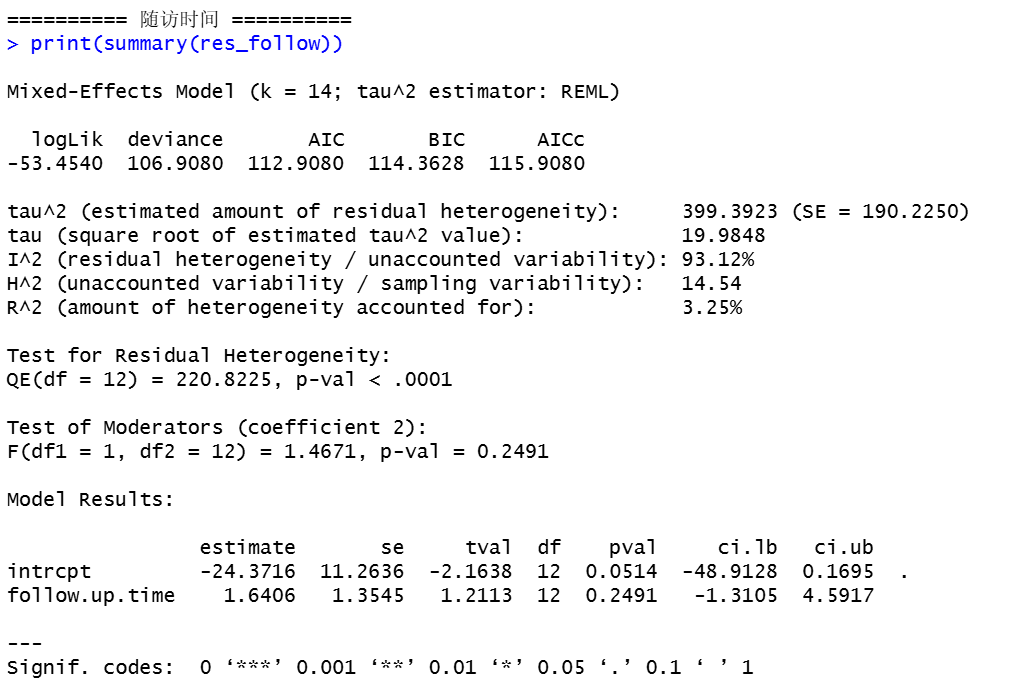


**S26 Fig.** Univariate meta- regression for BOP(male propotion)


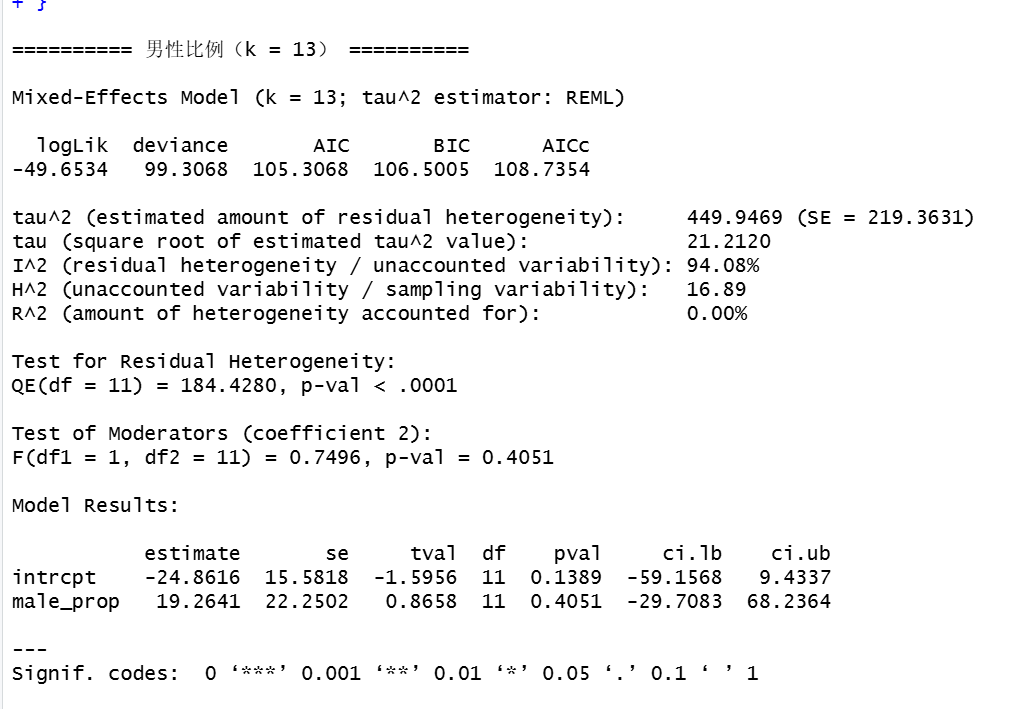


**S27 Fig.** Univariate meta- regression for BOP(sample)


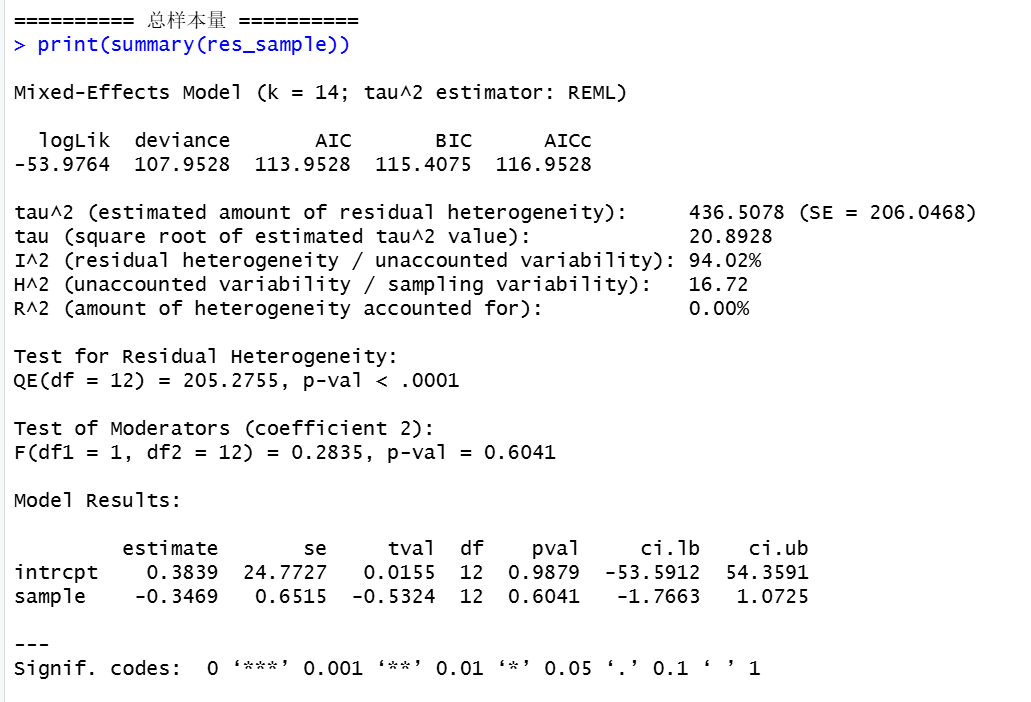


**S28 Fig.** Univariate meta- regression for BOP(publication year)


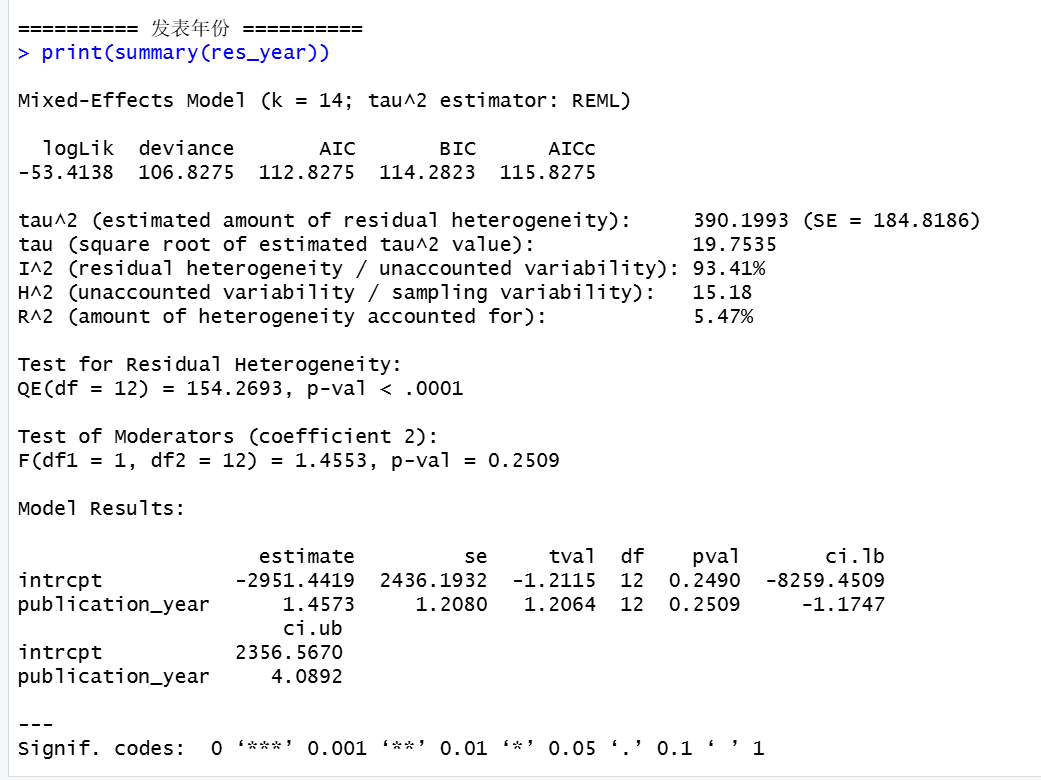


**S29 Fig.** Univariate meta- regression for PPD(age)


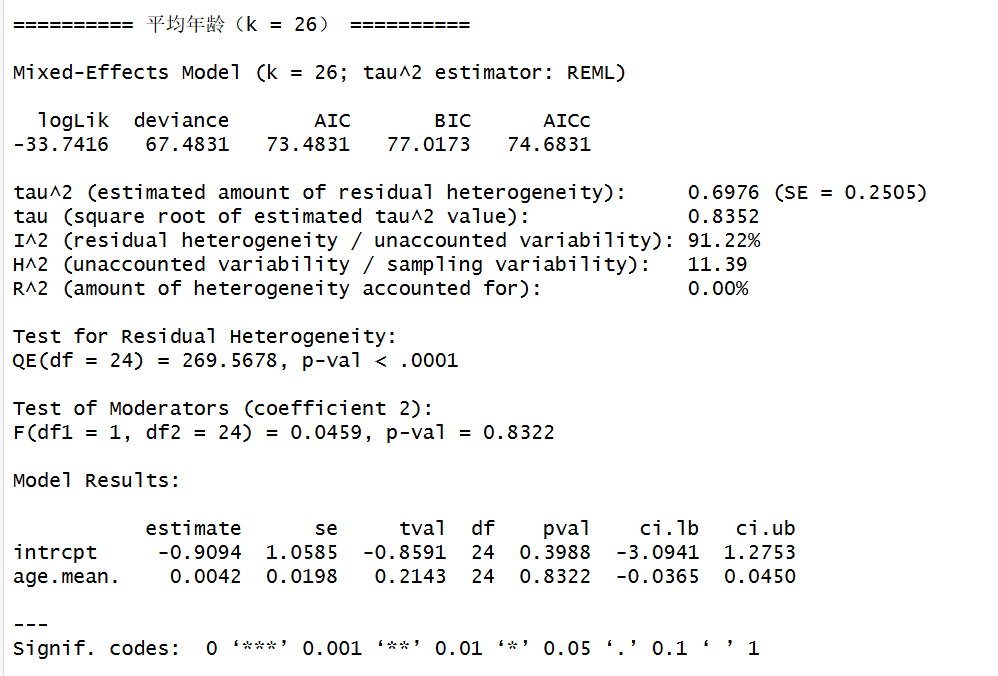


**S30 Fig.** Univariate meta- regression for PPD(country)


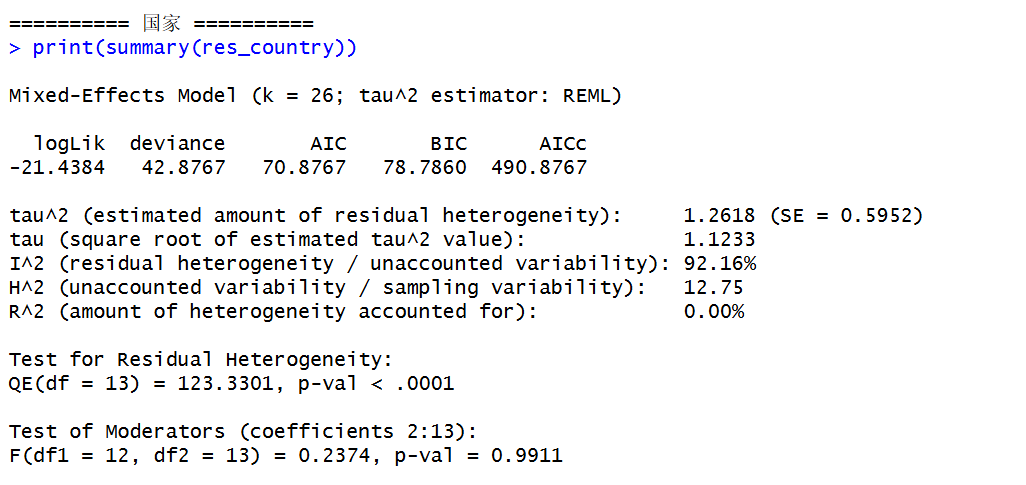


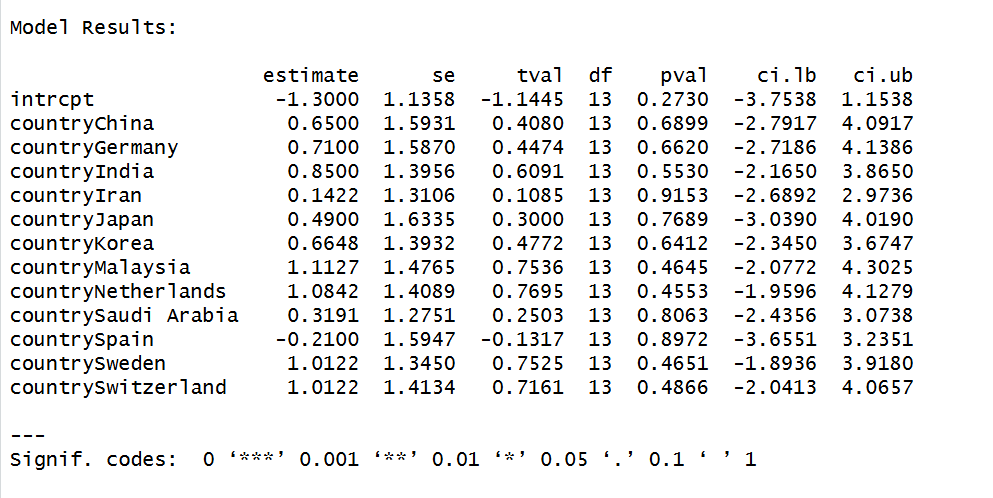


**S31 Fig.** Univariate meta- regression for PPD(follow-up time)


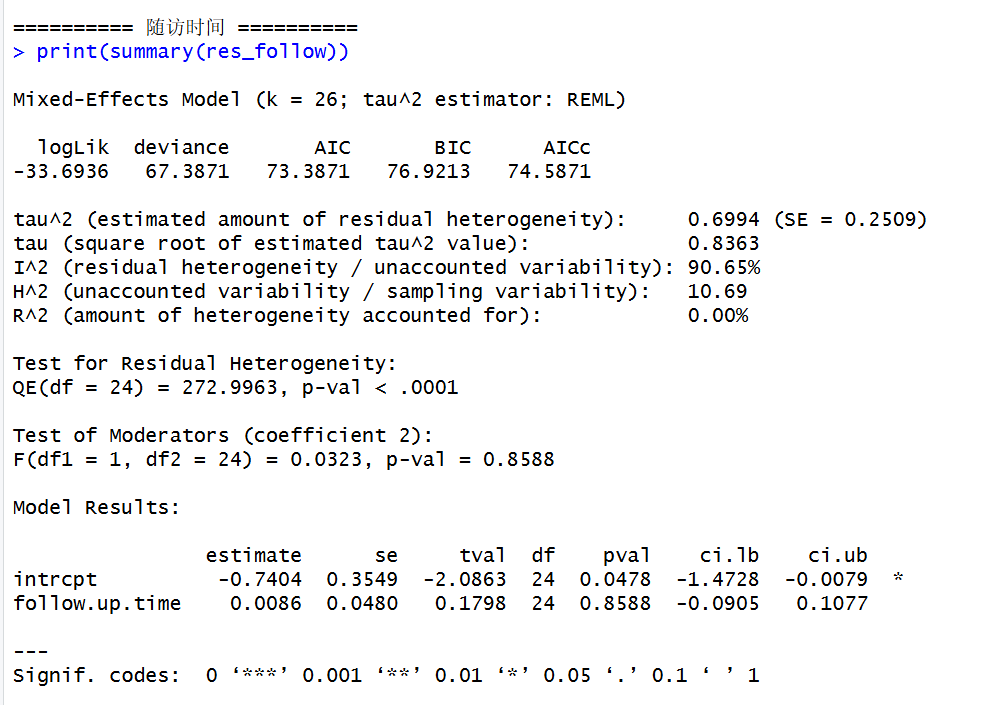


**S32 Fig.** Univariate meta- regression for PPD(male propotion)


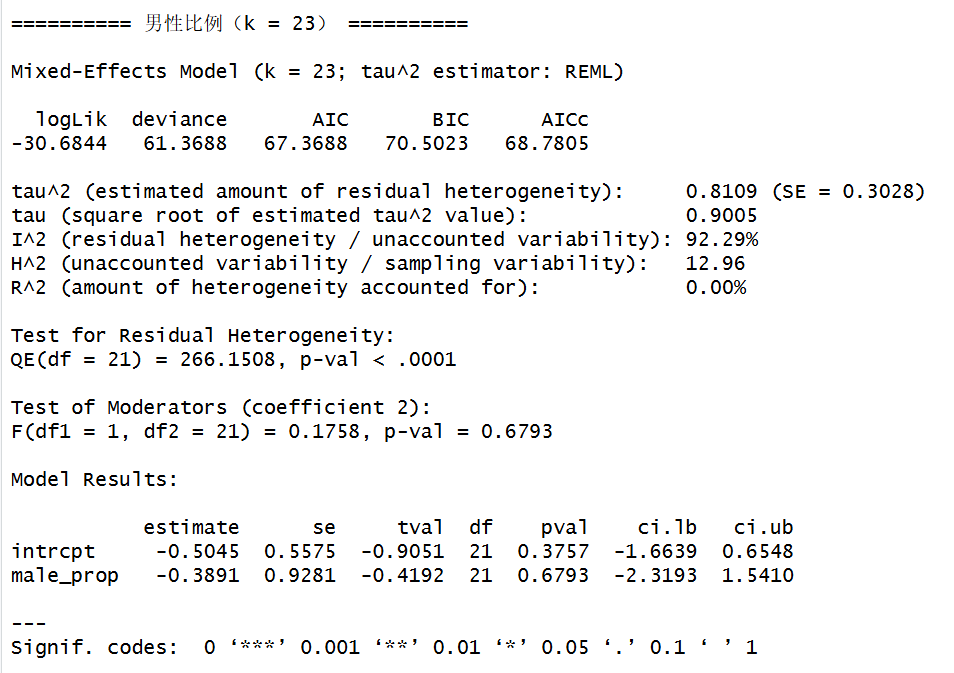


**S33 Fig.** Univariate meta- regression for PPD(sample)


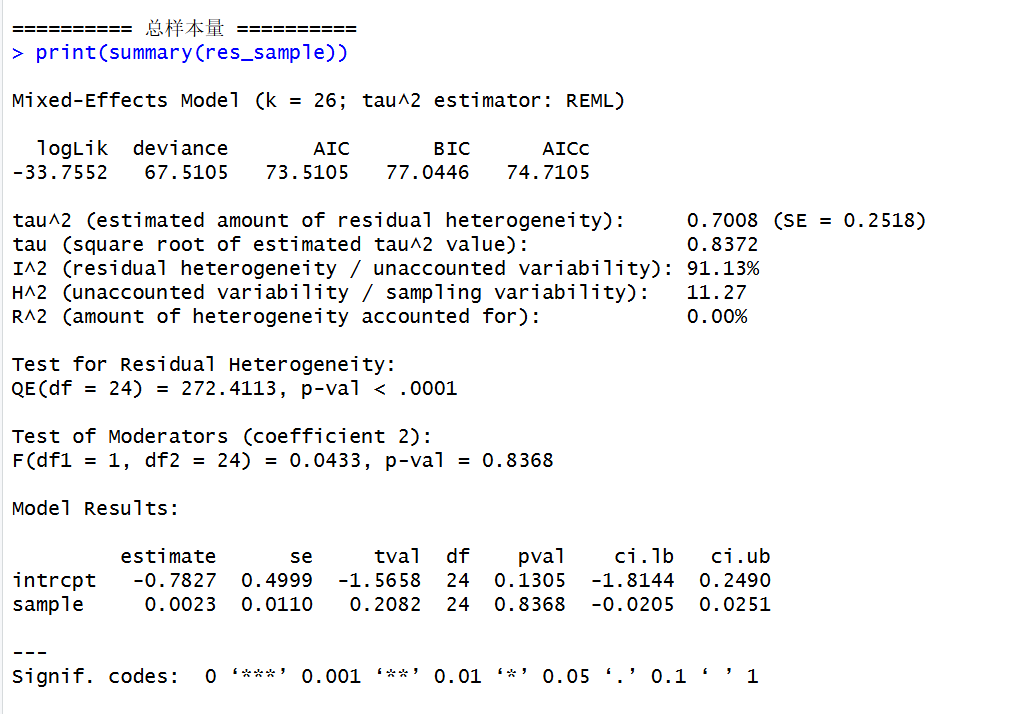


**S34 Fig.** Univariate meta- regression for PPD(publication year)


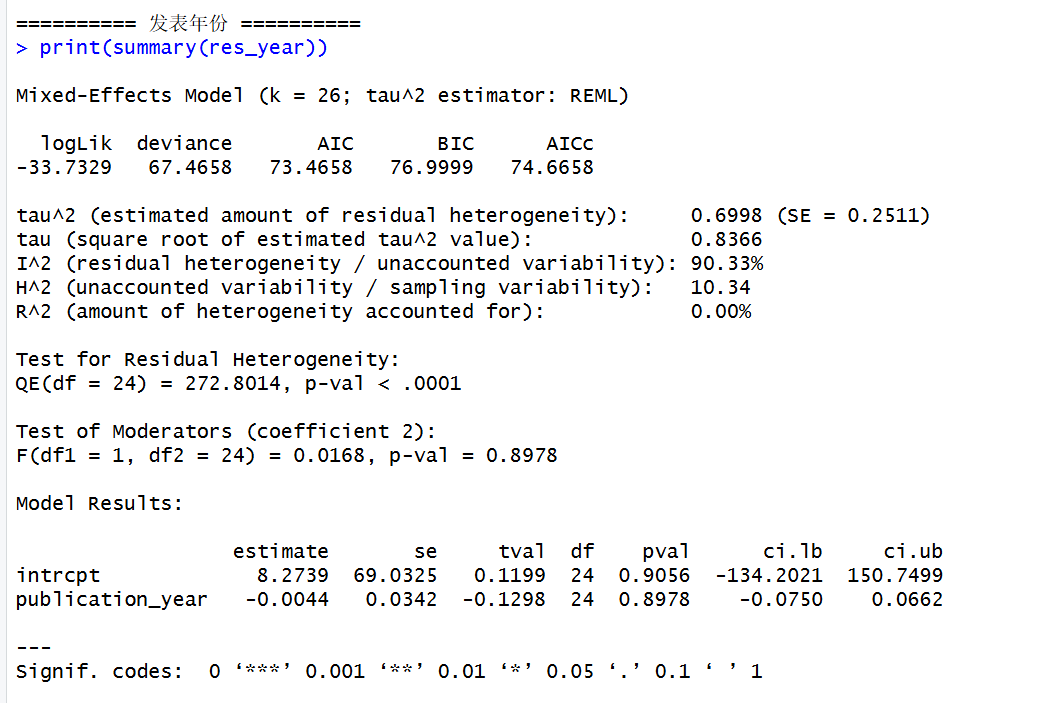


**S35 Fig.** Sensitivity analysis of probing pocket depth (PPD)


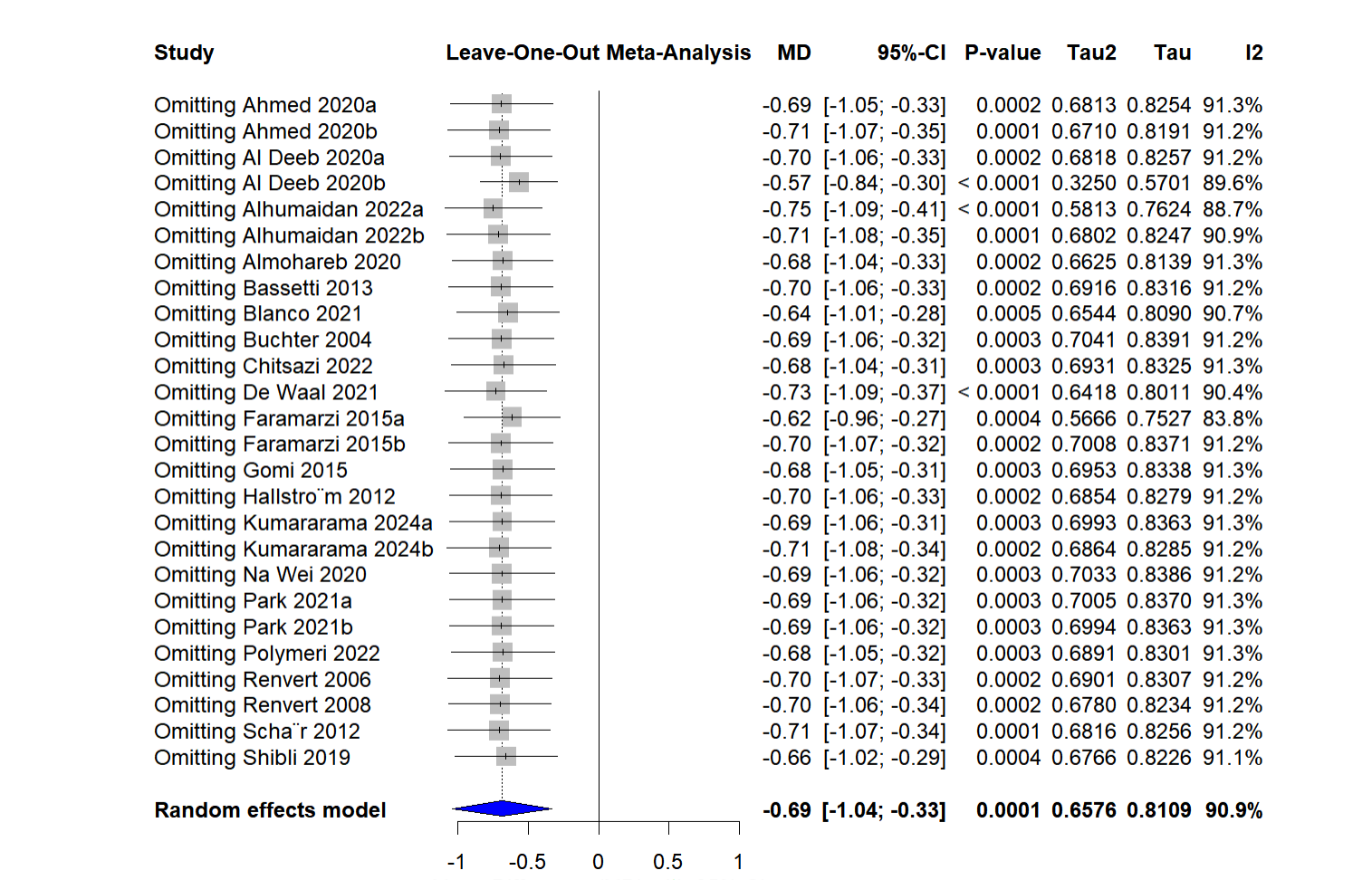


**S36 Fig.** Sensitivity analysis of bleeding on probing (BOP)


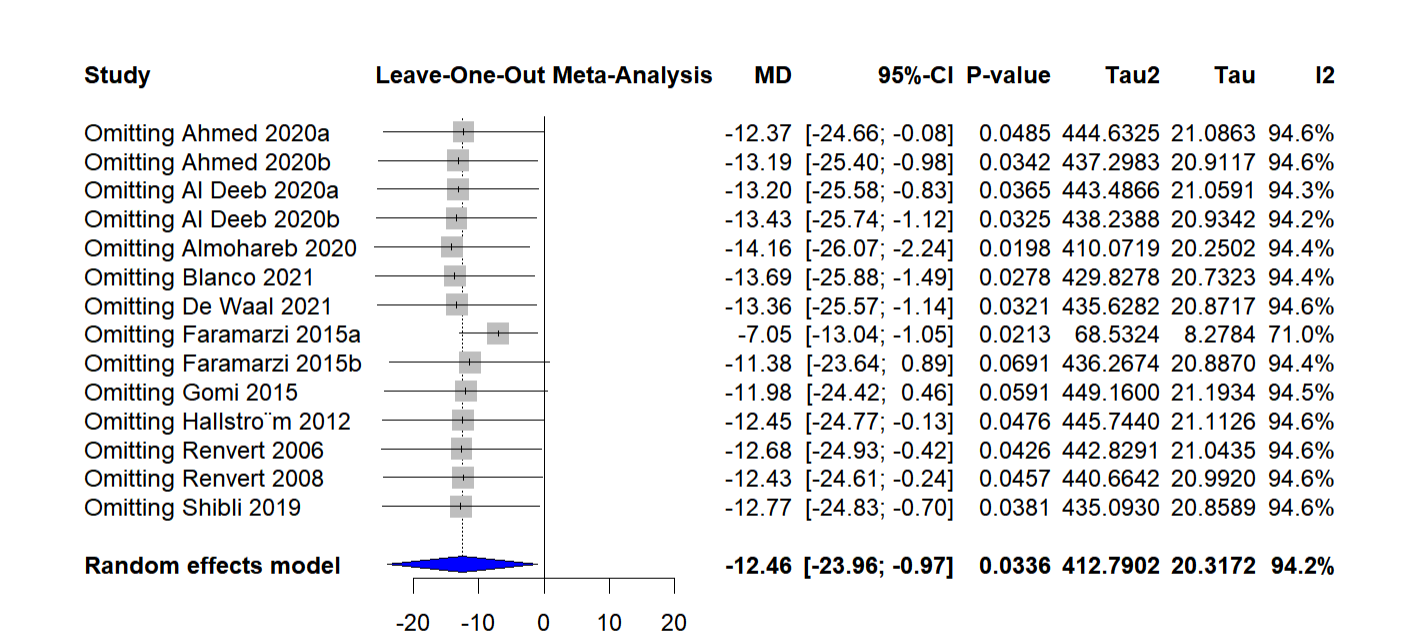


**S37 Fig.** Sensitivity analysis of clinical attachment level (CAL)


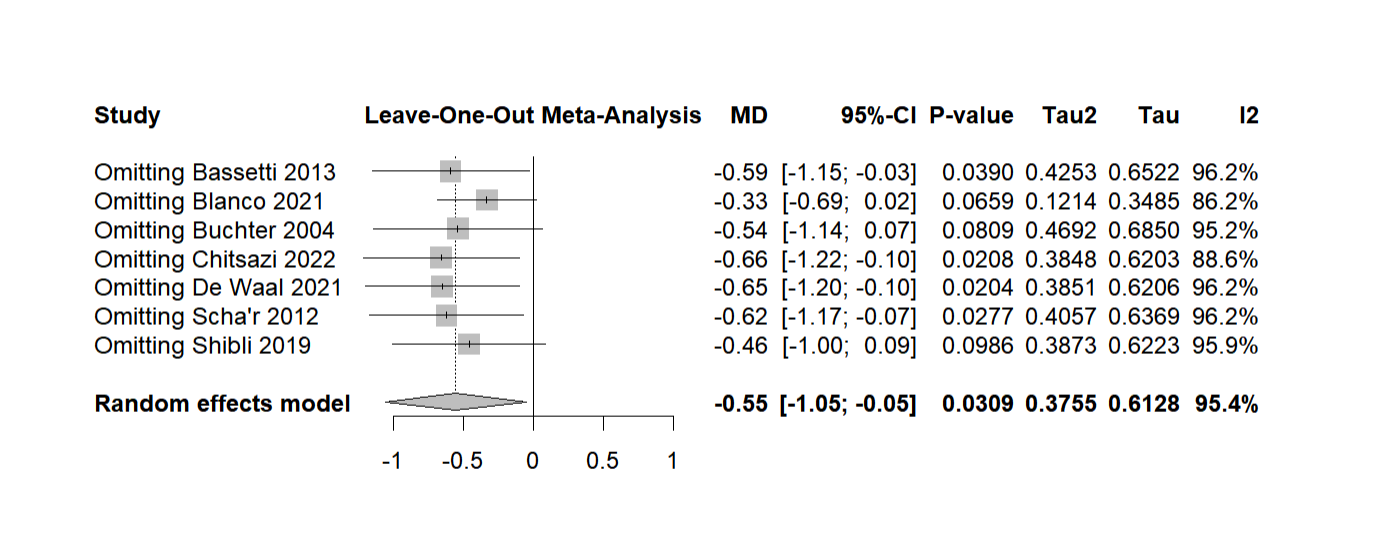


**S38 Fig.** Publication bias of probing pocket depth (PPD)


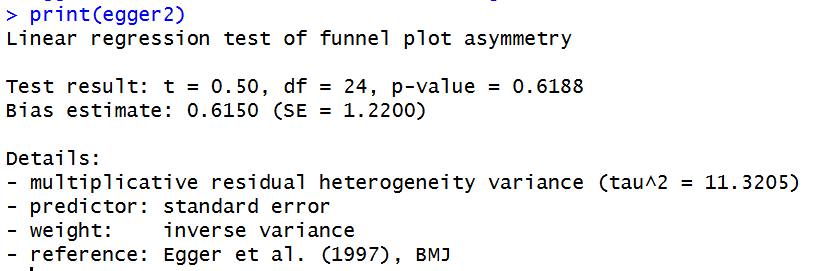


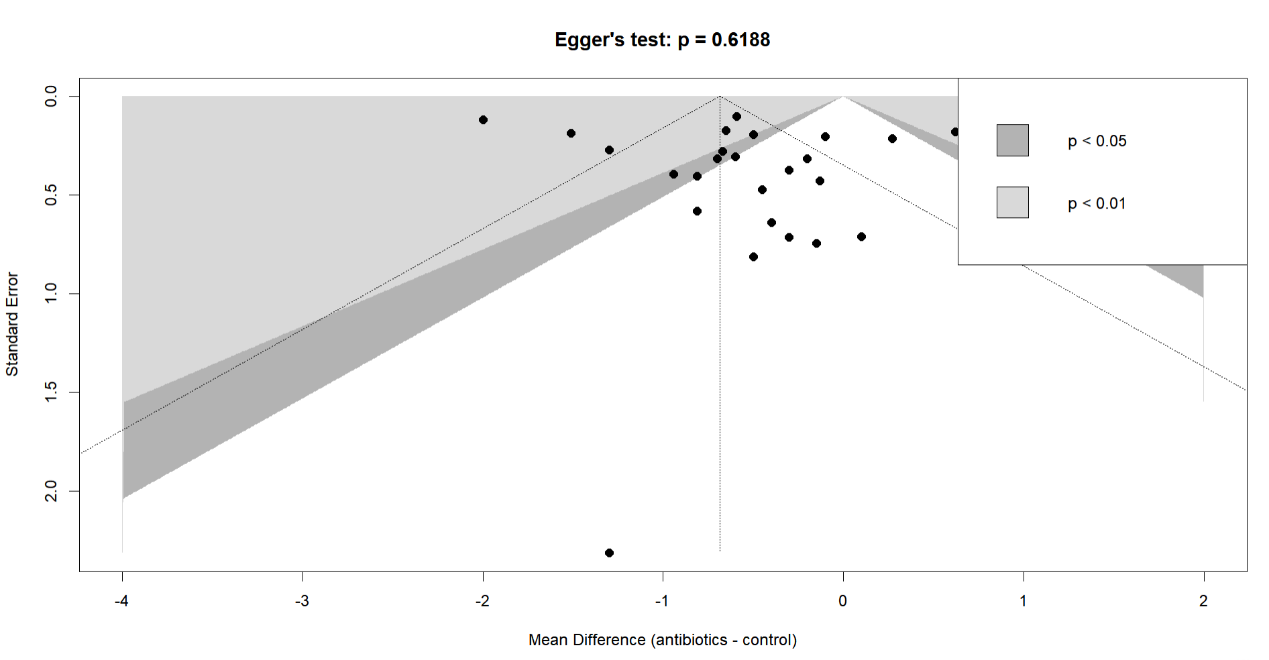


**S39 Fig.** Publication bias of bleeding on probing (BOP)


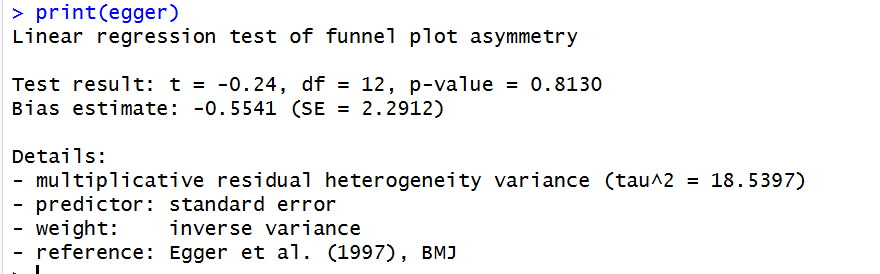


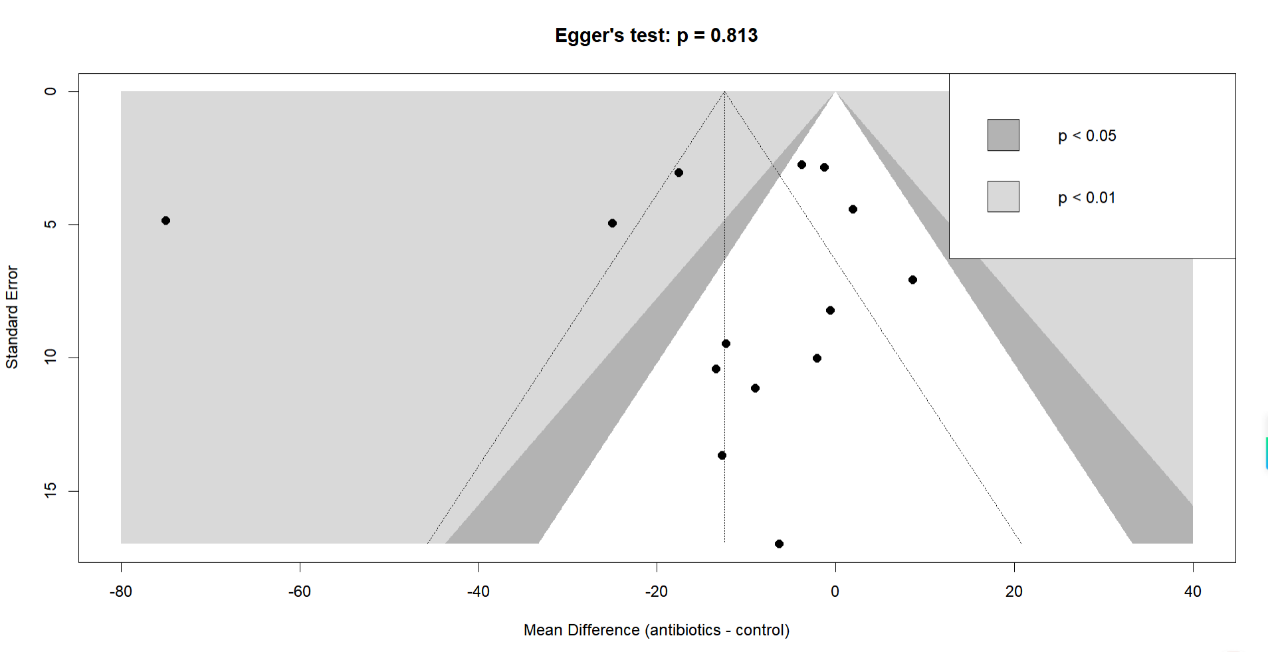


**S40 Fig.** Publication bias of plaque score (PS)


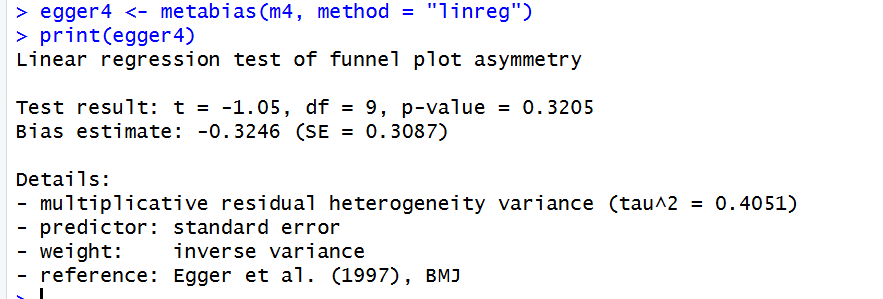


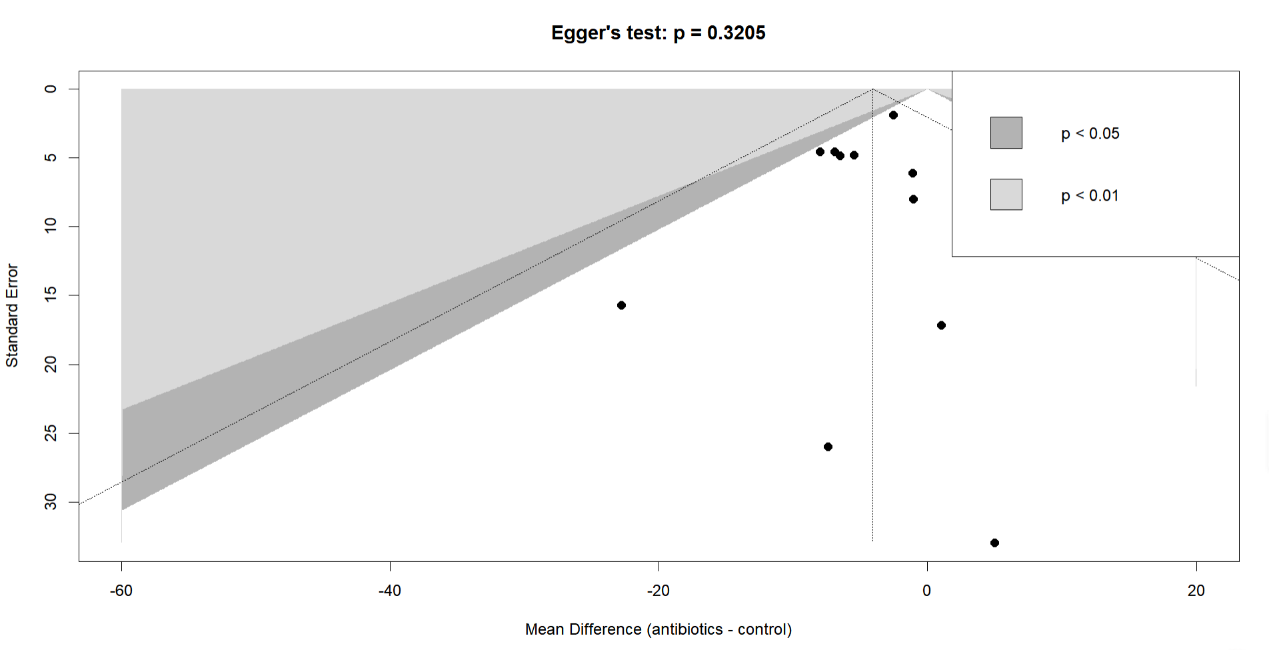

Supplement: S1 File — (ZIP) [file pone.0352311.s001.zip › Supplementary Materials.docx]
